# Purification, Structural Characterization, and Genomic Insights of an Anti-MRSA Peptide from Mangrove-Derived *Brevibacillus* sp. A34

**DOI:** 10.3390/life16101697

**Published:** 2026-10-10

**Authors:** Thanyamai Thongin, Namfa Sermkaew, Apichart Atipairin, Sucheewin Krobthong, Chanat Aonbangkhen, Jumpei Uchiyama, Nuttapon Songnaka

**Affiliations:** 1 School of Pharmacy, Walailak University, Thasala, Nakhon Si Thammarat 80160, Thailand; thanyamai.tho@wu.ac.th (T.T.); namfa.se@wu.ac.th (N.S.); apichart.at@wu.ac.th (A.A.); 2 Drug and Cosmetics Excellence Center, Walailak University, Thasala, Nakhon Si Thammarat 80160, Thailand; 3 Center of Excellence in Natural Products Chemistry (CENP), Department of Chemistry, Faculty of Science, Chulalongkorn University, Bangkok 10330, Thailand; sucheewin.k@chula.ac.th (S.K.); chanat.a@chula.ac.th (C.A.); 4 Center of Excellence on Petrochemical and Materials Technology, Chulalongkorn University, Pathumwan, Bangkok 10330, Thailand; 5 Department of Infectious Diseases, Graduate School of Medicine, Dentistry and Pharmaceutical Sciences, Okayama University, Okayama 700-8558, Japan; uchiyama@okayama-u.ac.jp

**Keywords:** *Brevibacillus* sp., antimicrobial peptide, AMP, mangrove, genome, anti-MRSA

## Abstract

The widespread prevalence of methicillin-resistant *Staphylococcus aureus* (MRSA) in community and healthcare settings has driven the urgent search for novel antibacterial agents. Mangrove sediments are an underexplored source of antimicrobial-producing bacteria. This study characterized an antimicrobial fraction and the genome of the mangrove-derived isolate *Brevibacillus* sp. A34. The active fraction was purified via ammonium sulfate precipitation, cation-exchange chromatography, and reversed-phase chromatography. De novo MS/MS analysis assigned the octapeptide LLLLLLAH (905.19 Da), designated as A34 AMP, which was further confirmed by chemical synthesis. This active peptide inhibited all four tested *S. aureus* strains, including three MRSA strains, with a minimum inhibitory concentration (MIC) of 62 µg/mL. At 2× and 4× MIC, A34 AMP exhibited a concentration-dependent bactericidal effect within 20 h. Increased SYTOX Green uptake and morphological alterations supported a membrane permeabilization mechanism, and antimicrobial activity was retained following thermal, pH, protease, and surfactant treatments. Genome mining identified 23 putative biosynthetic gene clusters, including an 11-module nonribosomal peptide synthetase (NRPS) cluster linked to A34 AMP through three-dimensional adenylation-domain modeling. Collectively, these findings highlight A34 AMP from *Brevibacillus* sp. A34 as a promising bioactive anti-staphylococcal peptide scaffold.

## 1. Introduction

Antimicrobial resistance (AMR) represents a severe challenge to global health, compromising the efficacy of therapies for infectious diseases across human, veterinary, and environmental domains. Data from 2019 indicate that drug-resistant bacterial pathogens were directly responsible for 1.27 million fatalities while being broadly associated with an estimated 5 million deaths overall. The primary drivers of this mortality include treatment-resistant bacteremia, pneumonia, tuberculosis, and intra-abdominal infections [1]. The burden of these infections falls disproportionately on low- and middle-income countries (LMICs), driven by systemic vulnerabilities such as deficient surveillance networks, restricted access to novel therapeutics, and fragile healthcare infrastructures. Notably, resistance to standard drug classes, particularly beta-lactams and fluoroquinolones, is implicated in over 70% of these fatalities, demonstrating the critical necessity for comprehensive global interventions [2]. In the absence of effective countermeasures, predictive models suggest that AMR-related mortality could escalate to 10 million annual deaths by 2050, presenting profound risks to worldwide economic stability and public health systems [3].

Methicillin-resistant *Staphylococcus aureus* (MRSA) causes a diverse array of severe clinical conditions, including potentially fatal pneumonia, endocarditis, bacteremia, and complicated skin and soft-tissue infections [4]. The ubiquitous presence of this pathogen across both healthcare and community settings makes these infections notoriously challenging to manage. Strikingly, recent comprehensive analyses reveal that MRSA exhibited the greatest increase in attributable mortality burden between 1990 and 2021 [1]. In the 2024 update to the World Health Organization (WHO) Bacterial Priority Pathogens List, MRSA is designated as a high-priority threat [5]. Paradoxically, while public health frameworks classify MRSA as highly treatable due to the availability of alternative antibiotics and established decolonization protocols, it remains the single pathogen–drug combination responsible for the greatest health loss globally [1]. This profound and ongoing clinical burden exposes a critical vulnerability in current therapeutic arsenals, reinforcing the urgent demand for novel antibacterial substances that operate via distinct mechanisms of action compared to traditional antibiotics.

Antimicrobial peptides (AMPs) and other peptide-derived natural products represent one possible source of such compounds. AMPs form a diverse group and differ considerably in size, charge, hydrophobicity, three-dimensional structure, and target specificity. Many bacterial AMPs act at the cell envelope, where they may increase membrane permeability, alter the membrane potential, or interfere with cell-wall-associated processes. *Brevibacillin* produced by *Brevibacillus laterosporus*, for example, binds to lipoteichoic acid before disrupting the cytoplasmic membrane of *S. aureus* [6]. However, membrane disruption is not a universal mechanism, and antibacterial activity depends strongly on peptide structure and the physiology of the target organism. In addition, promising in vitro potency does not establish therapeutic potential by itself. Peptide stability, selectivity, mammalian-cell toxicity, production yield, and susceptibility to biological fluids must also be considered.

Mangrove sediments are a useful but still incompletely explored source of antimicrobial-producing microorganisms. Their microbial communities experience changing salinity, oxygen availability, nutrient conditions, and intense ecological competition [7,8,9]. Several bioactive peptide-producing bacteria have recently been isolated from mangrove sediments in the same geographical area examined in the present study. These include a bacitracin-like anti-MRSA peptide from *Bacillus paralicheniformis* NNS4-3 [10], an AMP active against *Pseudomonas aeruginosa* and *Klebsiella pneumoniae* from *Paenibacillus thiaminolyticus* NNS5-6 [11], and an anionic anti-MRSA peptide from *Virgibacillus chiguensis* FN33 [12]. These findings support further exploration of this mangrove area as a source of bacteria producing chemically and functionally diverse antimicrobial compounds.

Members of the genus *Brevibacillus* are also recognized producers of nonribosomal peptides and lipopeptides. Several compounds from *Brevibacillus laterosporus,* including brevibacillin, brevicidine, laterocidine, and bogorol derivatives, have exhibited antibacterial activity against clinically relevant and antibiotic-resistant bacterial pathogens [13,14,15,16]. Antimicrobial production has also been reported in *Brevibacillus halotolerans*. A marine sediment isolate, *Brevibacillus halotolerans* 7WMA2, produced partially purified antimicrobial material in the 5–10 kDa range with activity against both Gram-positive and Gram-negative bacteria [17]. Another strain, B-4359, contained numerous biosynthetic gene clusters and exhibited antagonistic activity against plant pathogens [18]. Nevertheless, the molecular identities of the active products from this species and the relationships between antibacterial phenotypes and predicted biosynthetic gene clusters remain insufficiently resolved.

In this study, a mangrove sediment-derived bacterial isolate, designated A34, was selected for its activity against *S. aureus* and MRSA strains. This study aimed to identify the isolate by whole-genome sequencing, examine antimicrobial production during cultivation, purify and characterize the active fraction, and determine its inhibitory and bactericidal activities. Time-kill analysis, scanning electron microscopy, and SYTOX Green uptake were used to investigate its antibacterial action, while physicochemical stability and genome mining were evaluated to assess its potential for further antimicrobial development.

## 2. Materials and Methods

### 2.1. Materials

Luria–Bertani (LB) broth and Mueller–Hinton (MH) agar were supplied by Titan Biotech Ltd., Rajasthan, India. Agarose, calcium chloride, and magnesium sulfate were procured from Bio Basic Inc., Markham, Ontario, Canada. Acetonitrile (ACN), methanol, and trifluoroacetic acid (TFA) were obtained from Merck KGaA, Darmstadt, Germany. *S. aureus* TISTR 517 and *E. coli* TISTR 887 were acquired from the Thailand Institute of Scientific and Technology Research (TISTR), Pathum Thani, Thailand.

### 2.2. Sampling Points and Bacterial Isolation

Sediment samples were collected from a mangrove forest at Laem Talumphuk, Nakhon Si Thammarat, Thailand. Approximately 100 g of sediment was taken from each sampling point, immediately placed on ice, and transported to the laboratory within 24 h. For bacterial isolation, 10 g of each sediment sample was suspended in 90 mL of sterile 0.9% NaCl solution and incubated at 37 °C with shaking at 150 rpm for 30 min. Serial dilutions of the suspension were prepared up to 10^−6^, and 100 µL aliquots from each dilution were aseptically spread onto MH agar. Plates were incubated at 37 °C for up to 7 days and examined daily for bacterial growth. Morphologically distinct colonies were selected and purified by repeated streaking on fresh MH agar to obtain pure isolates [19,20].

### 2.3. Screening for Antimicrobial Activity

Preliminary antimicrobial screening was performed using the cross-streak assay [20] and the agar well diffusion assay [21] against a panel of seven indicator bacteria selected to represent standardized Gram-positive and Gram-negative reference strains and clinical multidrug-resistant isolates. Specifically, the panel comprised methicillin-susceptible *Staphylococcus aureus* TISTR 517 and the Gram-negative reference strains *Escherichia coli* TISTR 887, *Pseudomonas aeruginosa* TISTR 357, and *Klebsiella pneumoniae* TISTR 1383—obtained from the Thailand Institute of Scientific and Technological Research (TISTR)—to represent major World Health Organization (WHO) priority pathogens with distinct cell-envelope architectures, and three methicillin-resistant *S. aureus* (MRSA) isolates (strains 142, 1096, and 2468) to evaluate efficacy against relevant resistant phenotypes. Briefly, test isolates were streaked as a single straight line on MH agar plates and incubated at 30 °C for 4 days. Indicator strains, including *S. aureus* TISTR 517, MRSA strains 142, 1096, and 2468, and *E. coli* TISTR 887, were adjusted to an optical density of 0.1 at 625 nm in sterile 0.9% NaCl and then streaked perpendicularly to the test isolates without direct contact. Plates were subsequently incubated at 37 °C for 16–18 h, and antimicrobial activity was assessed based on the presence of inhibition zones adjacent to the test isolates. Isolates showing strong inhibitory activity or activity against more than one indicator strain were selected for further evaluation by the agar well diffusion assay [21].

For the agar well diffusion assay, selected isolates were cultured in MH broth at 30 °C with shaking at 150 rpm for 4 days. Cell-free supernatants were obtained by centrifugation at 10,000× *g* at 4 °C for 15 min and tested against the indicator strains on MH agar plates. Each well was loaded with 100 µL of supernatant, while sterile MH broth served as the negative control. After incubation at 37 °C for 16–18 h, antimicrobial activity was determined by measuring the diameter of the clear zones. All experiments were performed in triplicate, and the results were expressed as the mean ± standard deviation (SD).

### 2.4. Production Kinetics of Antimicrobial Compounds

For the evaluation of the time course of antimicrobial production, the bacterial isolate demonstrating the strongest inhibitory activity in the preliminary agar well diffusion screen was selected for kinetic profiling. An inoculum was prepared by suspending an isolated colony in sterile 0.9% (*w*/*v*) NaCl to an optical density at 625 nm (OD_625_) of 0.1, which was subsequently transferred at a 1% (*v*/*v*) ratio into 50 mL of Mueller–Hinton (MH) broth. Cultivation was carried out at 30 °C under orbital agitation at 150 rpm over a 168 h period. Culture aliquots were withdrawn at 4 h intervals throughout the initial 24 h and at 24 h intervals thereafter until 168 h. At each sampling point, biomass accumulation was tracked spectrophotometrically via OD_625_ measurements, after which cell-free supernatants (CFSs) were harvested by centrifugation (15,000× *g*, 4 °C, 10 min) and evaluated for antibacterial activity using the agar well diffusion method [22].

### 2.5. Purification of the Antimicrobial Fraction

The isolate exhibiting the most potent antimicrobial activity during initial screening was designated as strain A34. Strain A34 was cultivated in MH broth under previously optimized conditions for antimicrobial production. Following cultivation, bacterial cells were removed by centrifugation at 10,000× *g* and 4 °C for 30 min, and the resulting CFS was subjected to sequential ammonium sulfate fractionation at 25%, 50%, and 75% saturation. The precipitate from each step was recovered by centrifugation at 18,000× *g* and 4 °C for 15 min and resuspended in 50 mM ammonium acetate buffer (pH 5.0) containing 50 mM NaCl. Each preparation was subsequently desalted by dialysis against 50 mM ammonium acetate buffer (pH 5.0) at 4 °C for 16 h using a 3.5 kDa molecular-weight cut-off membrane. Finally, antibacterial activity in each dialyzed fraction was assessed against MRSA strain 2468 using the agar well diffusion assay.

Subsequent purification of the bioactive dialysate was carried out via a modified two-stage chromatographic workflow [23]. Initially, the dialyzed material was fractionated by cation-exchange chromatography (CIEX) on an ÄKTA protein purification system (GE Healthcare Bio-Sciences AB, Uppsala, Sweden) fitted with an SP Sepharose Fast Flow column pre-equilibrated in 50 mM ammonium acetate buffer (pH 5.0). Retained species were desorbed across a 25 mL total elution volume using a linear salt gradient ranging from 0 to 1 M NaCl prepared in the equilibration buffer. The effluent was continuously monitored spectrophotometrically at 214 nm, and 1.5 mL fractions were recovered and assayed for inhibitory activity against MRSA strain 2468. Bioactive CIEX fractions were subsequently combined and further resolved by reversed-phase chromatography (RPC) on an Inertsil^®^ ODS-3 C18 column (4.6 × 250 mm; GL Sciences, Tokyo, Japan) operated at a constant flow rate of 1 mL/min. Chromatographic separation employed a binary solvent system comprising 0.1% (*v*/*v*) trifluoroacetic acid (TFA) in water (eluent A) and 0.1% (*v*/*v*) TFA in 70% (*v*/*v*) aqueous acetonitrile (eluent B), programmed as follows: isocratic elution at 0% eluent B for 25 min, a linear gradient from 0% to 60% eluent B over 120 min, and a final hold at 100% eluent B for 40 min. Peaks were recorded at 214 nm and fractionated into 1.5 mL aliquots. Prior to testing for anti-MRSA (strain 2468) activity, the collected RPC fractions were dried under vacuum and redissolved in purified water to match their initial collection volume.

### 2.6. Mass Spectrometric Characterization and De Novo Peptide Sequencing

De novo peptide sequencing was performed by LC–MS/MS according to a previously described method [23]. An UltiMate 3000 LC system coupled to a high-resolution mass spectrometer (Thermo Fisher Scientific, Waltham, MA, USA) was used for the analysis. The sample was dissolved in an aqueous solution containing 0.1% formic acid and 1% acetonitrile, and a 3 µL aliquot was injected onto an analytical C18 column. Chromatographic separation was achieved using a 40 min gradient between mobile phase A (0.1% formic acid in water) and mobile phase B (0.1% formic acid in acetonitrile) at a flow rate of 300 µL/min. Solvent blanks containing 0.1% formic acid were analyzed between samples to minimize carryover. Electrospray ionization settings were 3.2 kV and 300 °C.

Full-scan mass spectra were acquired over an *m*/*z* range of 400–2200 at a resolution of 120,000, with an automatic gain control (AGC) target of 1 × 10^6^ and a maximum injection time of 100 ms. The full-scan data were processed using FreeStyle software (version 1.4), with the PPD peak-detection algorithm and a signal-to-noise threshold of 5, to generate a precursor-ion inclusion list. The sample was subsequently reanalyzed under identical chromatographic conditions in parallel reaction monitoring (PRM) mode. Tandem mass spectra were acquired at a resolution of 30,000, with an AGC target of 5 × 10^5^, a maximum injection time of 150 ms, and an *m*/*z* isolation window of 1.4.

The PRM spectra were analyzed using PEAKS Studio X (Bioinformatics Solutions Inc., Waterloo, ON, Canada) for de novo sequence assignment. Candidate peptide sequences were retained when both sequence and confidence scores exceeded 70. The theoretical molecular mass, isoelectric point, and grand average of hydropathicity (GRAVY) were calculated from the candidate sequence using ProtParam on the ExPASy server. To validate the identity and antibacterial activity of the identified sequence, the candidate octapeptide (LLLLLLAH) was custom-synthesized by U2Bio Co., Ltd. (Bangkok, Thailand) via standard 9-fluorenylmethoxycarbonyl (Fmoc) solid-phase peptide synthesis (SPPS). After stepwise chain elongation and acidolytic cleavage from the resin support, the crude synthetic product was purified by preparative reversed-phase high-performance liquid chromatography (RP-HPLC). Chemical homogeneity (≥95% purity) was verified by analytical RP-HPLC on a YMC-Triart C18 column (4.6 × 250 mm, 3 µm particle size) using a 30 µL injection volume. Chromatographic analysis was performed at a constant flow rate of 1 mL/min with spectrophotometric detection at 214 nm, employing a binary mobile-phase system composed of 0.1% (*v*/*v*) TFA in water (eluent A) and 0.1% (*v*/*v*) TFA in acetonitrile (eluent B). The elution program consisted of a linear gradient from 0% to 50% eluent B over 50 min, followed by an immediate step transition to 100% eluent B at 50.01 min, which was held until run completion at 60 min. The molecular mass and primary sequence fidelity of the purified synthetic peptide were subsequently confirmed by liquid chromatography–mass spectrometry [24].

### 2.7. Determination of Minimum Inhibitory Concentration (MIC) and Minimum Bactericidal Concentration (MBC) Values

The antimicrobial potency of the purified peptide was evaluated by determining the MIC and MBC using the broth microdilution method in accordance with the Clinical and Laboratory Standards Institute (CLSI) guidelines [25]. Indicator strains were cultured on MH agar at 37 °C for 16–18 h. Single colonies were suspended in cation-adjusted Mueller–Hinton broth (CAMHB) and adjusted to an optical density of 0.1 at 625 nm (approximately 1 × 10^8^ CFU/mL); the suspension was then diluted 1:20 in CAMHB to obtain the working inoculum. Two-fold serial dilutions of the RPC purified fraction and chemically synthesis of A34 peptide were prepared in CAMHB in 96-well plates, yielding final peptide concentrations ranging from 0.5 to 250 µg/mL. A 10 µL aliquot of the working inoculum was added to each well, yielding a final inoculum of 5 × 10^5^ CFU/mL. Wells containing inoculum without peptide and wells containing only CAMHB served as negative and blank controls, respectively. The optical density at 625 nm was recorded at 4 h intervals up to 24 h using a microplate reader. The MIC was defined as the lowest peptide concentration that restricted bacterial growth to ≤10% of that of the negative control. To determine the MBC, aliquots from wells at and above the MIC were subcultured onto MH agar and incubated at 37 °C for 48 h; the lowest concentration showing no visible colony formation was recorded as the MBC. All assays were performed in triplicate, and median MIC and MBC values are reported.

### 2.8. Time-Kill Assay

Time-kill kinetics were evaluated using a modified CLSI M26-A protocol [26]. Mid-exponential-phase cultures of the test strains were adjusted to approximately 5 × 10^5^ CFU/mL in CAMHB and exposed to the purified peptide at 0.5×, 1×, 2× and 4× MIC; an antibiotic-free culture served as the growth control. Cultures were incubated at 37 °C with shaking at 150 rpm, and 100 µL aliquots were sampled at 0, 2, 4, 6, 8, 12 and 24 h. Each aliquot was serially diluted ten-fold in 0.9% NaCl, plated on MH agar, and incubated at 37 °C for 18–24 h before colony enumeration. The results were expressed as log_10_ CFU/mL versus time, and bactericidal activity was defined as a ≥3 log_10_ reduction in viable counts relative to the initial inoculum.

### 2.9. Bacterial Membrane Permeability Assay

The effect of the purified peptide on bacterial membrane integrity was assessed using the membrane-impermeant nucleic acid-binding dye [27,28]. Test strains were cultured on MH agar at 37 °C for 16–18 h. Single colonies were suspended in 0.9% NaCl, centrifuged at 10,000× *g* for 5 min, and washed three times with the same solution to remove residual culture medium. The washed cells were resuspended in 0.9% NaCl and adjusted to an optical density of 0.1 at 625 nm (OD_625_). Aliquots (95 µL) of the bacterial suspension were transferred to a black 96-well microplate. A 5 µL aliquot of a SYTOX Green working solution prepared in DMSO was then added to each well to achieve a final dye concentration of 2.5 µM in the completed 200 µL assay mixture. The plate was incubated in the dark at room temperature for 15 min to allow for equilibration of the dye. The purified peptide was subsequently added to achieve final concentrations equivalent to 0.25×, 0.5×, 1×, 2×, and 4× MIC, and the final volume of each well was adjusted to 200 µL. Triton X-100 at a final concentration of 1% (*v*/*v*) was used as the positive control for membrane disruption, whereas sterile deionized water, serving as the peptide vehicle, was used as the negative control. Baseline fluorescence was recorded before peptide addition. Membrane permeabilization was monitored by fluorescence emission arising from SYTOX Green binding to intracellular DNA (excitation 504 nm; emission 523 nm). Fluorescence was recorded at 0, 30 and 60 s; 5, 10, 15, 30 and 60 min; and 2, 3, 6, 12, 18 and 24 h. Experiments were performed in triplicate, and mean fluorescence values were plotted against time to characterize membrane disruption kinetics.

### 2.10. Morphological Alterations Examined by Scanning Electron Microscopy (SEM)

Peptide-induced changes in bacterial surface morphology were examined by SEM using a modified procedure described previously [29,30]. Test strains were cultured on MH agar at 37 °C for 16–18 h. Bacterial colonies were suspended in CAMHB, adjusted to an optical density of 0.1 at 625 nm, corresponding to approximately 1 × 10^8^ CFU/mL, and subsequently diluted 1:20 in CAMHB. Aliquots (10 µL) of the diluted bacterial suspension were mixed with 90 µL of peptide solutions prepared in CAMHB to achieve final peptide concentrations equivalent to the respective MIC and MBC, yielding a final bacterial inoculum of 5 × 10^5^ CFU/mL. Untreated bacterial suspensions served as negative growth controls, whereas CAMHB without bacterial cells or peptide served as the sterility blank. Samples were incubated at 37 °C and collected immediately after peptide addition (0 h) and after 1, 3, and 18 h of exposure. At each time point, bacterial cells were harvested by centrifugation at 10,000× *g* for 15 min and washed three times with sterile 0.9% (*w*/*v*) NaCl by centrifugation at 10,000× *g* for 5 min per wash. The cell pellets were fixed with 2.5% (*v*/*v*) glutaraldehyde prepared in 0.1 M phosphate buffer (pH 7.2) at 4 °C for 48 h. The fixed cells were dehydrated through a graded ethanol series ranging from 20% to 100% (*v*/*v*), critical-point dried using CO_2_, and sputter-coated with gold. The specimens were subsequently examined using a scanning electron microscope, and representative micrographs were acquired at a nominal magnification of 20,000× [31].

### 2.11. Stability Assessment of the AMP

The thermal and pH stabilities, protease sensitivity, and surfactant compatibility of the purified peptide were evaluated using a modified procedure described previously [32]. To determine thermal stability, peptide solutions were incubated at 4, 40, 60, 80, and 100 °C for 1 h or autoclaved at 121 °C for 10 and 15 min. After cooling to room temperature, residual antibacterial activity was determined against the indicator strains and compared with an untreated control maintained at 37 °C. To evaluate pH stability, peptide solutions were adjusted to pH 1.0–14.0 using 1 N HCl or 1 N NaOH and incubated at 37 °C for 2 h. The samples were subsequently neutralized to pH 8.0, and residual activity was compared with a control maintained at pH 8.0. Protease sensitivity was assessed by incubating the peptide at 10× MIC with proteinase K, trypsin, or α-chymotrypsin solutions (1 mg/mL) prepared in Tris–HCl buffer at their respective optimal pH values of 7.0, 8.0, and 8.2. Reactions were incubated at 37 °C for 1 h and terminated by heating at 80 °C for 10 min, and evaluated for residual activity relative to an enzyme-free buffer control. Surfactant compatibility was evaluated by mixing the peptide at 10× MIC with sodium dodecyl sulfate (SDS) or Triton X-100 at a final concentration of 1 mg/mL. A peptide solution containing water instead of surfactant served as the control. All stability assays were performed in triplicate. Inhibition-zone diameters were recorded, and residual activity was expressed as relative activity (mean ± SD) compared with the corresponding control. Statistical significance was assessed using a Student’s *t*-test, with *p* < 0.05 considered statistically significant.

### 2.12. Whole-Genome Sequencing and Genomic Analysis

To identify the producer strain, a single colony grown on MHA at 30 °C for 7–14 days was used for genomic DNA extraction with the GenElute™ Bacterial Genomic DNA Kit (Sigma-Aldrich, Warren, MI, USA) according to the manufacturer’s instructions. Following genomic DNA extraction, the isolate underwent whole-genome sequencing by U2Bio Co., Ltd. (Seoul, Republic of Korea), using an Illumina HiSeq system (Illumina, San Diego, CA, USA) to produce 150 bp paired-end reads. All downstream bioinformatic analyses were carried out on the Galaxy Australia server (version 23.1) [33]. FastQC (version 0.12.1) [34] was used to evaluate sequence quality both pre- and post-trimming, whereas Fastp (version 0.23.4) [35] was employed to remove adapters and filter out low-quality reads (under 30 bp). De novo genome assembly was accomplished through the Shovill pipeline (version 1.1.0) integrated with SPAdes (version 3.15.5) [36]. The resulting assembly was then evaluated for overall completeness and quality using QUAST (version 5.2.0) [37] and CheckM (version 1.0.18) [38].

Taxonomic classification utilized a two-pronged strategy. Initially, the Genome BLAST Distance Phylogeny (GBDP) algorithm (accessed on 20 June 2026) [39] was applied against the TYGS database to assess genomic relatedness, facilitating the generation of a phylogenetic tree with pertinent reference strains to establish evolutionary relationships. To verify identification at the species level, FastANI (version 1.1.0) [40] was used to compute the Average Nucleotide Identity (ANI) between the FNL62 isolate and its nearest phylogenetic relatives. This sequence homology was then visually represented via the Proksee platform (https://proksee.ca, accessed on 20 June 2026). Functional annotation and coding sequence (CDS) identification were conducted using the NCBI Prokaryotic Genome Annotation Pipeline (PGAP) version 6.11 [41,42]. AntiSMASH (version 8.0.4) [43] was used to mine the annotated genome for biosynthetic gene clusters (BGCs). These BGC profiles were subsequently compared with known sequences from the GenBank [44] and MIBiG (version 4.0) [45] databases. Ultimately, the Proksee platform’s CGViewBuilder (version 2.0) was used to produce a comprehensive circular map of the FNL62 genome (https://proksee.ca, accessed on 20 June 2026), displaying its annotated features and genetic architecture within color-coded concentric tracks [46]. The antimicrobial resistance genes were screened using the Resistance Gene Identifier from the Comprehensive Antibiotic Resistance Database. The analysis was performed using the nucleotide assembly, the default curated identity thresholds, and a minimum reference-sequence coverage of 50% [47].

### 2.13. Statistical Analysis

All experiments were performed in triplicate, and results are expressed as the mean ± standard deviation (SD). Statistical significance was evaluated using a Student’s t-test or one-way analysis of variance (ANOVA), where *p* < 0.05 was considered statistically significant.

## 3. Results

### 3.1. Screening, Selection, and Preliminary Characterization of Isolate A34

Bacterial isolates were recovered from mangrove sediment collected at Laem Talumphuk, Nakhon Si Thammarat Province, Southern Thailand. Preliminary screening using the cross-streak assay against MRSA strain 2468 identified several isolates exhibiting antibacterial activity. The isolates were then evaluated via the agar well diffusion assay against a broader indicator comprising *S. aureus* TISTR 517, MRSA strains 142, 1096, and 2468, *E. coli* TISTR 887, *P. aeruginosa* TISTR 357, and *K. pneumoniae* TISTR 1383. Among the isolates tested, isolate A34 produced the largest inhibition zones, successfully inhibiting all four Gram-positive strains. However, no inhibition was observed against the three Gram-negative species. Consequently, isolate A34 was therefore selected for further study.

### 3.2. Growth Kinetics and Antimicrobial Production Profile

**Figure 1:**
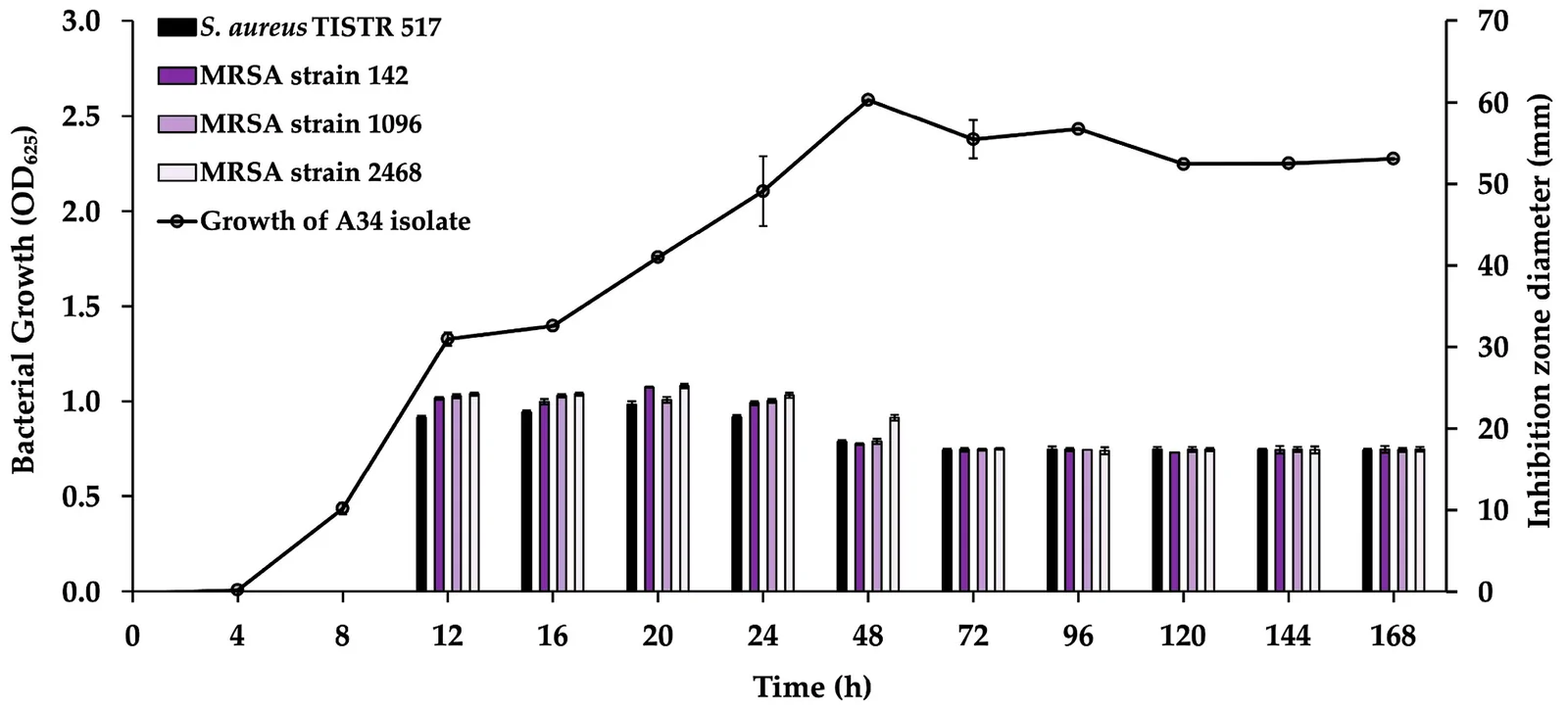
Growth and antibacterial production kinetics of isolate A34 over a 168 h cultivation period. Bar graphs represent inhibition zone diameters against the four indicator strains, and the line represents cell growth measured as OD_625_. Data are presented as the mean ± SD.

The growth of isolate A34 and concomitant production of antimicrobial activity were monitored over 168 h in MH broth (Figure 1). Following an initial 4 h lag phase, cell density increased throughout the exponential phase and reached a maximum OD_625_ of 2.58 at 48 h. The culture then entered the stationary phase, which continued until 168 h. Antimicrobial activity in the CFS was evaluated against *S. aureus* TISTR 517 and three MRSA strains. Activity was first detected at 12 h, during the exponential phase, and remained high between 12 and 24 h. During this period, inhibition-zone diameters ranged from 21.30 ± 0.23 to 22.90 ± 0.40 mm against *S. aureus* TISTR 517, 23.10 ± 0.23 to 25.00 ± 0.06 mm against MRSA strain 142, 23.40 ± 0.25 to 24.00 ± 0.20 mm against MRSA strain 1096, and 24.10 ± 0.32 to 25.20 ± 0.26 mm against MRSA strain 2468. Although biomass continued to increase and peaked at 48 h, antimicrobial activity did not increase proportionally. Inhibition zone diameters remained relatively stable after 24 h and then declined gradually, although activity was still measurable at 168 h. All four strains exhibited similar temporal profiles. However, MRSA strain 2468 generally exhibited slightly larger inhibition zones, particularly between 20 and 48 h, indicating somewhat greater susceptibility under these assay conditions.

### 3.3. Whole-Genome Sequencing and Genomic Analysis of the Selected Isolate

**Figure 2:**
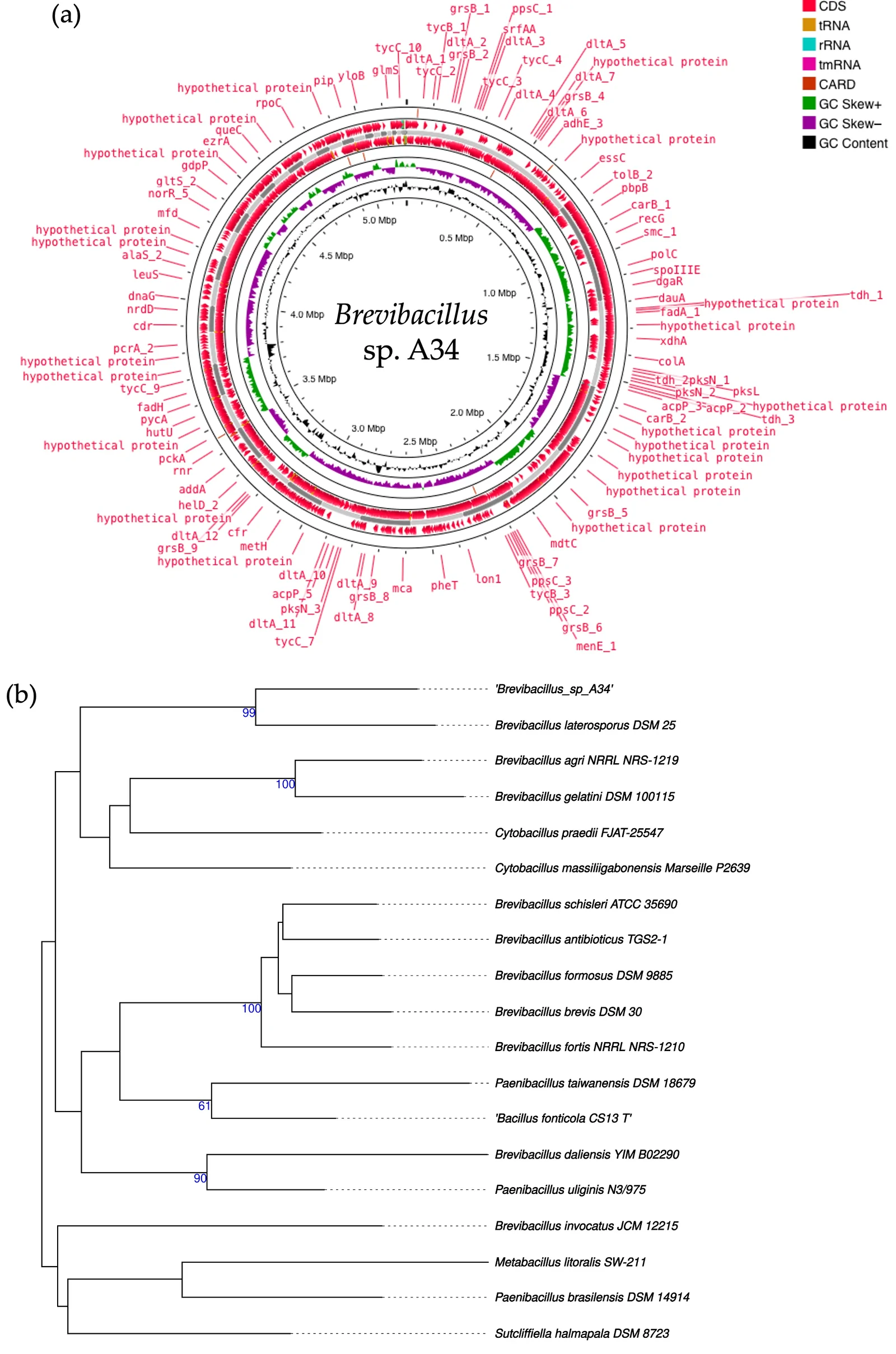
Genomic circular map and phylogenetic tree of strain A34. (**a**) Circular genome map showing coding sequence (CDS) annotations and gene predictions generated by Prokka. (**b**) Whole-genome-based phylogenetic tree showing the placement of strain A34 within the genus *Brevibacillus.* A34 forms a sister lineage to *Brevibacillus laterosporus* DSM 25, with 99% bootstrap support (values are shown at the corresponding nodes). The tree was inferred from genome sequence data using the TYGS platform.

The draft genome of strain A34 comprised 39 scaffolds totaling 5,169,413 bp (approximately 5.17 Mb), with a GC content of 41.23% and an N50 value of 261,405 bp. CheckM2 estimated 99.98% completeness and 0.02% contamination, indicating a high-quality assembly. Genome annotation predicted 4435 coding sequences, 54 tRNA genes, 3 rRNA genes, 8 ncRNA genes, and one tmRNA gene. No CRISPR arrays were detected (Figure 2a).

In the whole-genome-based phylogenetic tree, strain A34 was placed within the genus *Brevibacillus* and formed a well-supported sister lineage to *Brevibacillus laterosporus* DSM 25 (bootstrap value, 99%). This clade was distinct from other *Brevibacillus* species included in the analysis, such as *Brevibacillus agri*, *Brevibacillus gelatini*, *Brevibacillus schisleri*, *Brevibacillus formosus*, and *Brevibacillus brevis*. Thus, the phylogenomic analysis supported the placement of A34 within a lineage closely related to *Brevibacillus laterosporus* (Figure 2b).

### 3.4. Purification and Characterization of the Antimicrobial Compound

**Figure 3:**
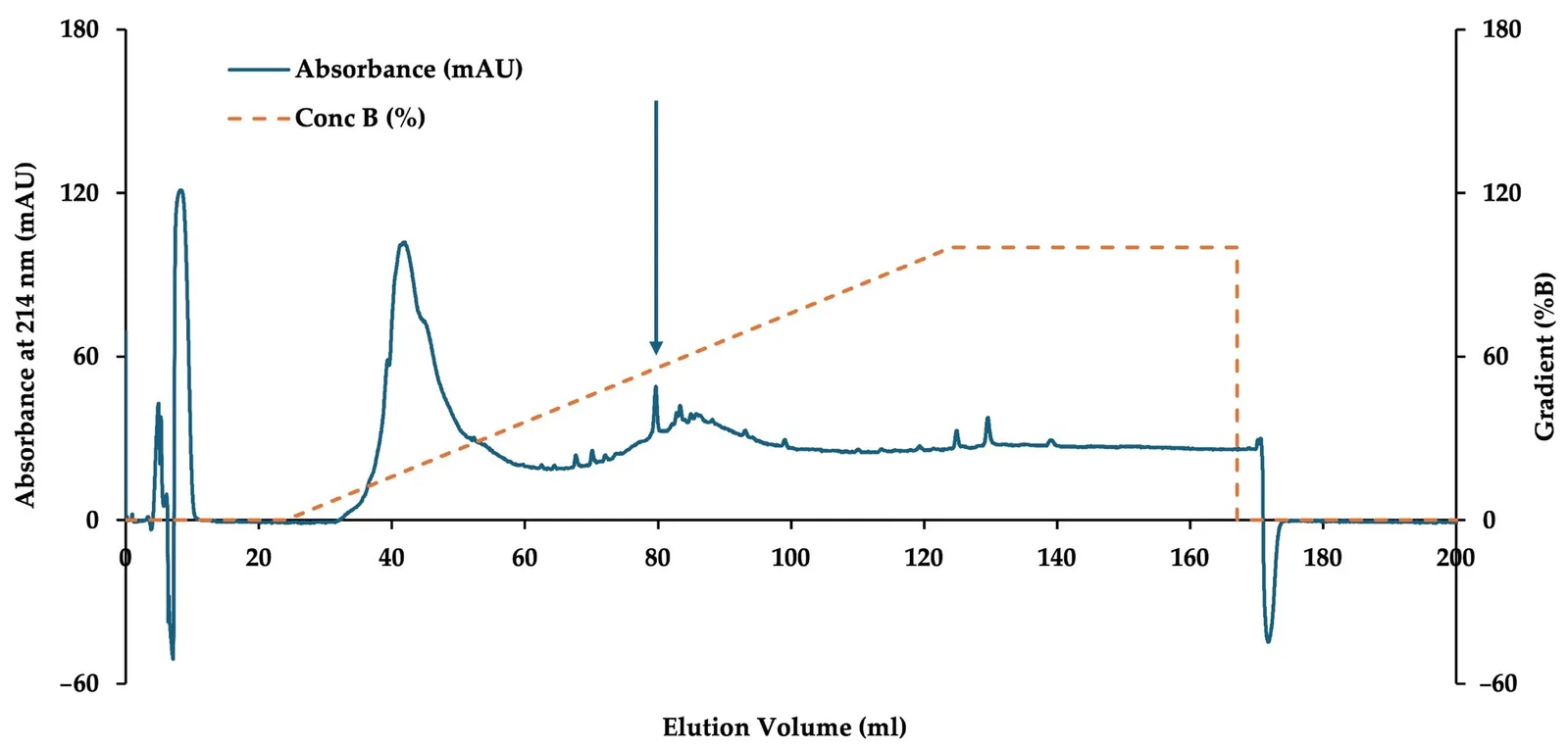
Reversed-phase chromatography (RPC) purification profile of A34 AMP. The chromatogram displays UV absorbance at 214 nm (left y-axis) and the mobile phase B (0.1% TFA in 70% acetonitrile; right y-axis) linear gradient plotted against elution volume (x-axis). The arrow indicates the active fraction exhibiting antibacterial activity against the indicator strain.

The antimicrobial compound produced by isolate A34 was partially purified via a three-step process ammonium sulfate precipitation, cation-exchange chromatography (CIEX), and reversed-phase chromatography (RPC). The active RPC fraction eluted at approximately 40% of the gradient (Figure 3). The purification yield, specific activity, and purification factor are summarized in Table 1, demonstrating that specific activity increased with each purification step while total yield decreased.

**Figure 4:**
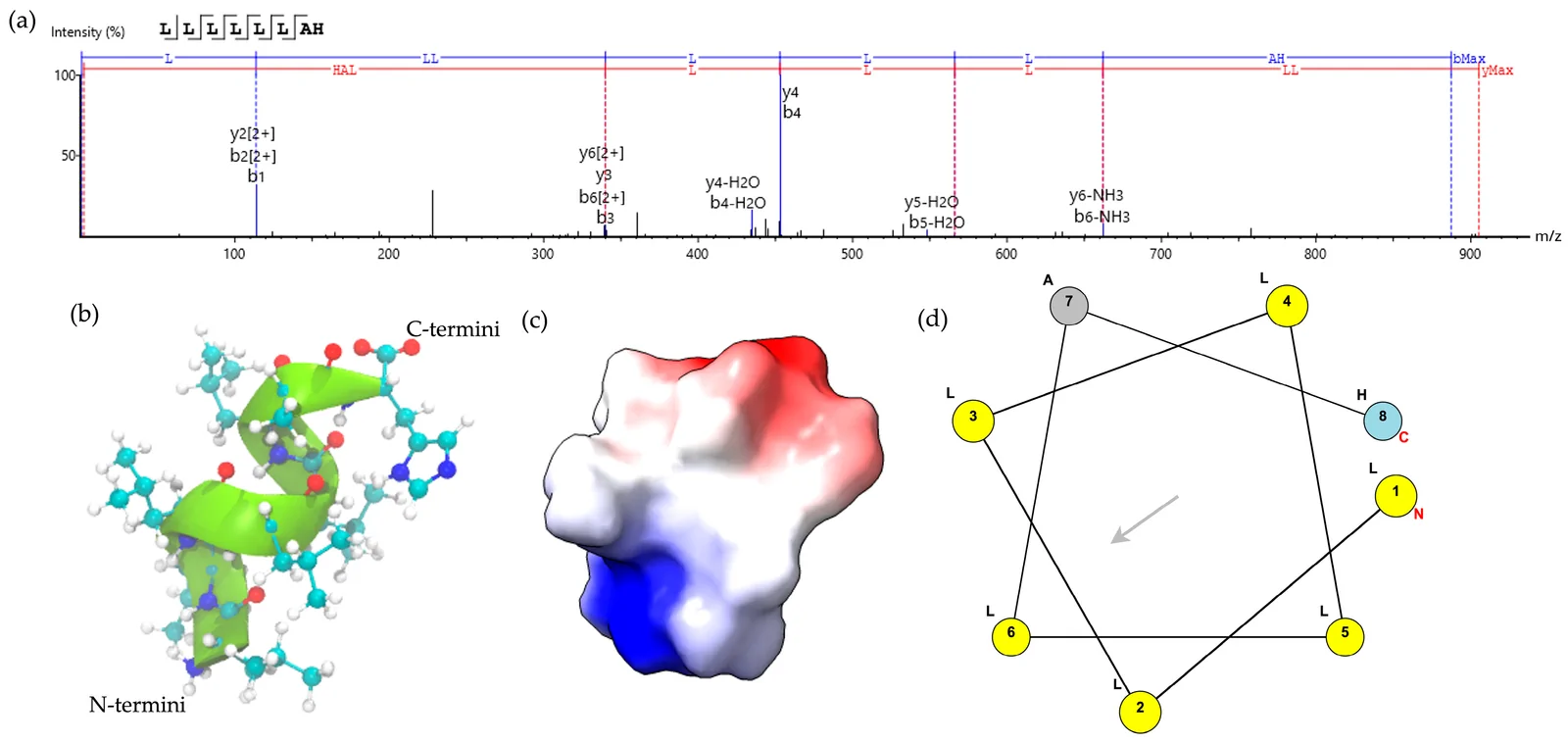
(**a**) De novo amino acid sequence of A34 AMP (LLLLLLAH) derived from LC–MS/MS fragmentation spectra. Dark blue and red lines (both solid and dotted) denote the experimentally observed b- and y-ion fragment masses, respectively. (**b**) Putative three-dimensional model predicted by AlphaFold (shown in CPK color representation), illustrating a short nascent α-helical segment. (**c**) Molecular surface electrostatic potential shaded in blue (positively charged), white (hydrophobic), and red (negatively charged). (**d**) Helical wheel projection of the modeled α-helical conformation. Circles numbered 1–8 represent the amino acid positions from the N-terminus to the C-terminus, with colors indicating chemical properties (yellow for hydrophobic leucine, grey for alanine, and light blue for histidine). The arrow indicates the direction of the net hydrophobic moment.

The RPC-purified A34-derived RPC fraction was subsequently analyzed by LC–MS/MS. Because standard tandem mass spectrometry cannot differentiate between the isobaric branched-chain isomers leucine and isoleucine, de novo interpretation of the LC–MS/MS fragmentation spectra initially indicated an octapeptide of the form [L/I]_6_AH, which was assigned as Leu-Leu-Leu-Leu-Leu-Leu-Ala-His (LLLLLLAH) in accordance with the leucine-specific adenylation domain predictions of the candidate biosynthetic gene cluster (Figure 4a). The predicted molecular mass was 905.1922 Da, and the theoretical isoelectric point (pI) was 7.81. Leucine accounted for 75.0% of the sequence, while alanine and histidine each accounted for 12.5%. The GRAVY score (2.675) and aliphatic index (305.0) were consistent with a highly hydrophobic, aliphatic peptide. Hydrophobic residues (Leu and Ala) constituted 87.5% of the sequence, and histidine, the only basic residue, accounted for the remaining 12.5%. The predicted net charge at pH 7.4 was +0.033, indicating that the peptide is essentially neutral under physiological conditions. In silico three-dimensional modeling by AlphaFold (accessed on 25 August 2026) predicted a short, nascent α-helical conformation spanning approximately two helical turns (Figure 4b), reflecting the high intrinsic helical propensity of the hexaleucine–alanine tract in a hydrophobic or membrane-bound environment. In this modeled conformation, the electrostatic potential surface displayed polar positive and negative regions at the N- and C-termini, respectively, whereas the central core remained strongly hydrophobic (Figure 4c). Correspondingly, the helical wheel projection illustrated a pronounced hydrophobic face opposite the polar terminal regions (Figure 4d). Based on its origin and antimicrobial properties, this peptide was designated as A34 AMP. To confirm the chemical identity of the de novo-assigned octapeptide and establish a chemically homogeneous reference standard for intrinsic bioactivity verification, the all-L octapeptide (NH_2_-Leu-Leu-Leu-Leu-Leu-Leu-Ala-His-COOH; LLLLLLAH) was chemically synthesized via Fmoc solid-phase peptide synthesis (SPPS). Analytical RP-HPLC separation confirmed that the purified synthetic A34 peptide achieved a chemical purity of 98.76% at 214 nm. Furthermore, electrospray ionization LC–MS analysis of the synthetic product confirmed an observed molecular mass matching the theoretical monoisotopic mass of native LLLLLLAH (905.19 Da), thereby validating the primary sequence integrity prior to comparative antimicrobial susceptibility testing.

### 3.5. Antimicrobial Activity of Purified Peptides

The antimicrobial activities of both the reversed-phase chromatography (RPC)-purified A34-derived RPC fraction (92.06% purity) and the chemically synthesized A34 peptide (98.76% purity) were evaluated against *S. aureus* TISTR 517 and three MRSA isolates (strains 142, 1096, and 2468) using broth microdilution minimum inhibitory concentration (MIC) and minimum bactericidal concentration (MBC) assays (Table 2). Based on the lyophilized dry weight of the RPC-purified material, the apparent MIC of the A34-derived RPC fraction was 62 µg/mL against all four tested strains, whereas the apparent MBC was 250 µg/mL against methicillin-susceptible *S. aureus* TISTR 517 and 62 µg/mL against each of the three MRSA strains. Notably, the chemically synthesized A34 peptide exhibited an identical susceptibility profile, yielding an intrinsic MIC of 62 µg/mL across all four strains, an MBC of 250 µg/mL against *S. aureus* TISTR 517, and a bactericidal MBC of 62 µg/mL (MBC/MIC ratio = 1) against all three MRSA strains (Table 2). The reference standard vancomycin exhibited MIC and MBC values of 2 µg/mL across all four strains. The complete concordance between the apparent activity of the RPC-purified fraction and the intrinsic activity of the synthetic octapeptide confirms that the LLLLLLAH sequence is the primary bioactive component responsible for the anti-staphylococcal and bactericidal activity of isolate A34.

### 3.6. Time-Kill Kinetics of A34 AMP

**Figure 5:**
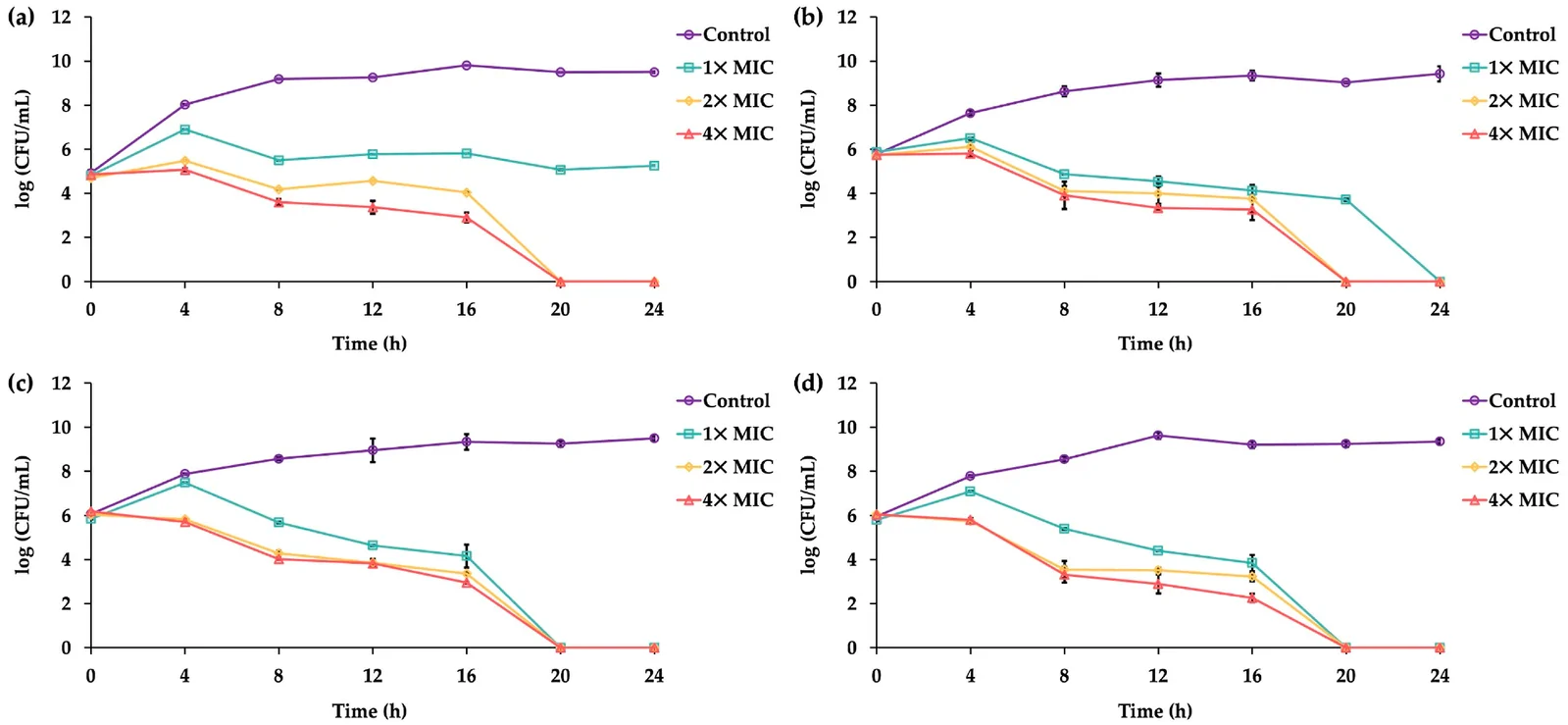
Time-kill curves of A34 AMP against (**a**) *S. aureus* TISTR 517, (**b**) MRSA strain 142, (**c**) MRSA strain 1096, and (**d**) MRSA strain 2468. Cells were exposed to A34 AMP at 1×, 2×, and 4× MIC for 24 h; untreated culture served as control. Viable colony counts are expressed as log CFU/mL. Values are presented as mean ± SD.

The bactericidal activity of A34 AMP against *S. aureus* TISTR 517 and three MRSA strains was evaluated using a time-kill assay at 1×, 2×, and 4× MIC, compared with an untreated control (Figure 5). Killing was rapid and concentration-dependent across all four strains. At 2×, and 4× MIC, viable counts fell below the limit of detection within 20 h. The estimated killing-rate constants at 2×, and 4× MIC were 0.055 and 0.140 h^−1^ for *S. aureus* TISTR 517 (Figure 5a); 0.152 and 0.186 h^−1^ for MRSA strains 142 (Figure 5b); 0.183 and 0.209 h^−1^ for MRSA strains 1096 (Figure 5c); and 0.197 and 0.262 respectively for MRSA strains 2468 (Figure 5d), respectively.

At 1× MIC, A34 AMP reduced viable counts by approximately 3 log_10_ CFU/mL across all four strains. The corresponding killing-rate constants were 0.018 h^−1^ for *S. aureus* TISTR 517 and 0.130, 0.156, and 0.166 h^−1^ for MRSA strains 142, 1096, and 2468, respectively. The lower value for *S. aureus* TISTR 517 indicated slower killing at 1× MIC than observed for the MRSA strains.

### 3.7. Morphological Alterations in Bacterial Cells Observed by SEM

**Figure 6:**
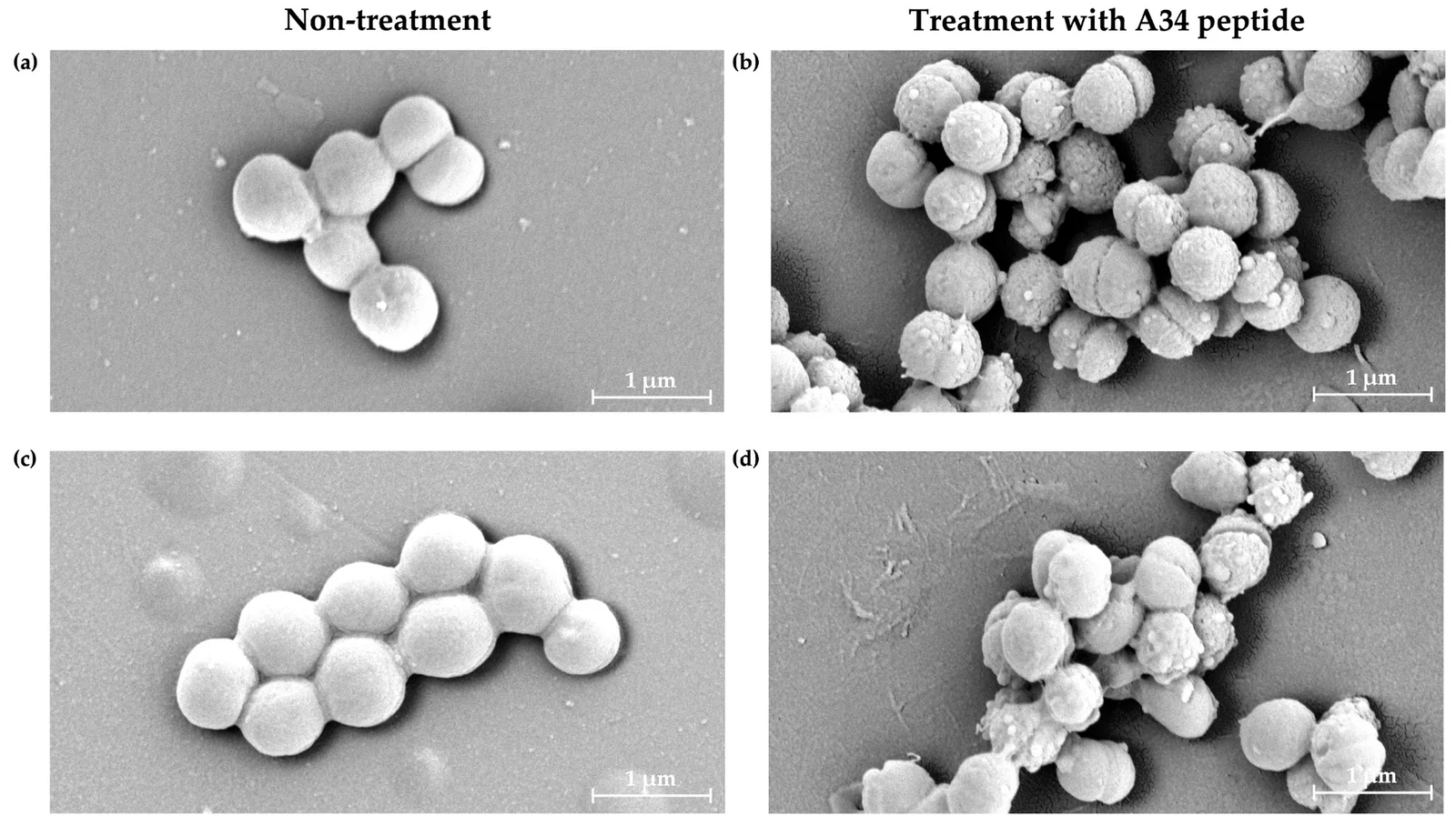
Scanning electron micrographs showing the effects of A34 AMP on (**a**,**b**) *S. aureus* TISTR 517 and (**c**,**d**) MRSA strain 2468. Untreated cells (**a**,**c**) had smooth, intact surface, whereas cells exposed to A34 AMP at 1× MIC (**b**,**d**) showed surface disruption and deformation. Images were obtained at 20,000× magnification.

The effects of A34 AMP at 1× MIC on the morphology of *S. aureus* and MRSA strain 2468 were examined by scanning electron microscopy (SEM) (Figure 6). Untreated cells of both strains showed the coccoid morphology typical of staphylococci, with smooth, intact surfaces. In contrast, cells exposed to A34 AMP had rough, irregular surfaces and prominent membrane blebs or vesicle-like structures. Many treated cells were also distorted or elongated and formed aggregates. These changes were consistent with disruption of the cell surface.

### 3.8. Effect of A34 AMP on Bacterial Membrane Permeability

**Figure 7:**
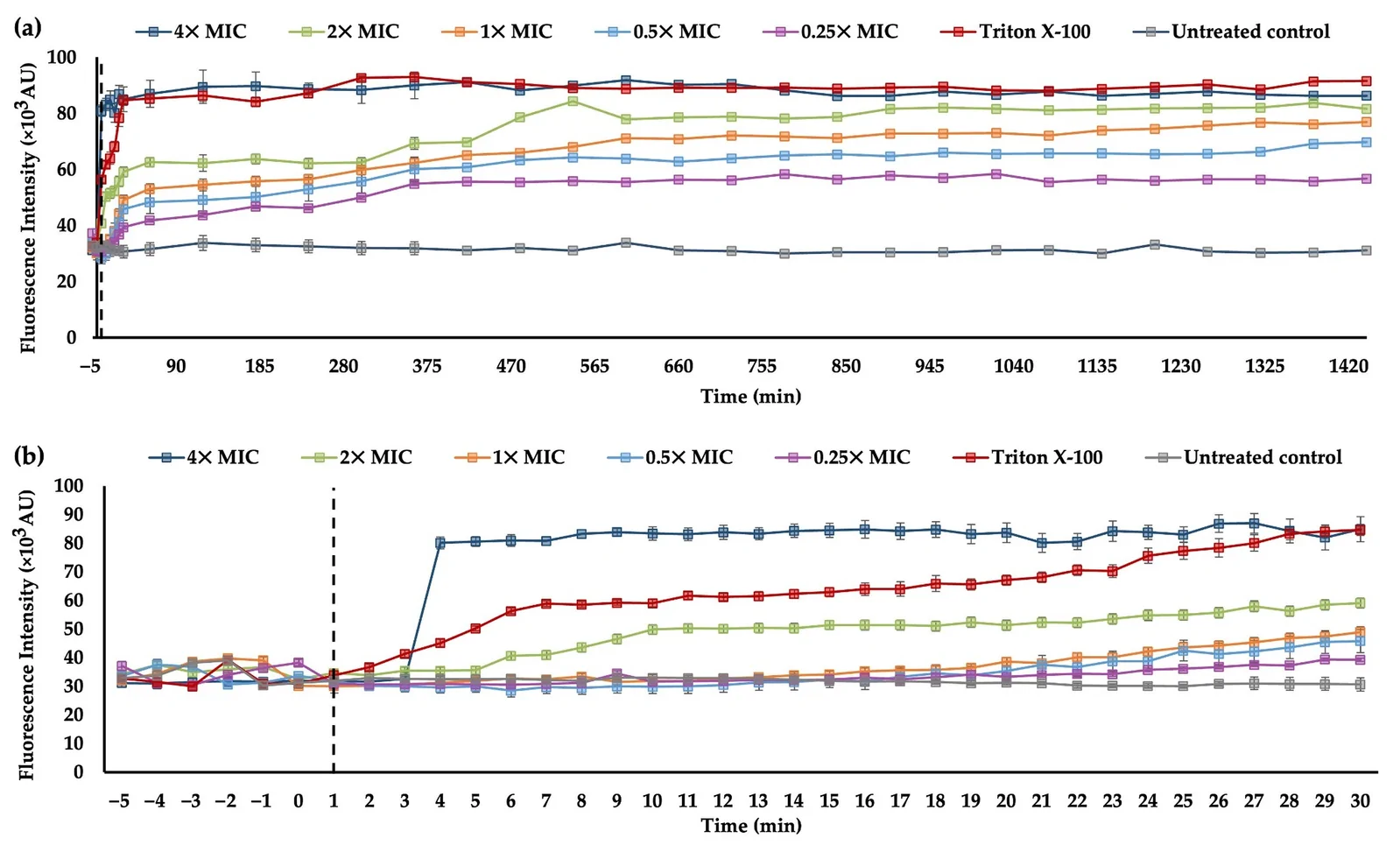
SYTOX Green fluorescence uptake by MRSA strain 2468 following exposure to AMP A34 at 0.25×, 0.5×, 1×, 2×, and 4× MIC. Triton X-100 (1%) and untreated cells served as the positive and negative controls, respectively. (**a**) Full 24 h (1420 min) kinetic profile of membrane permeabilization. (**b**) Expanded view of the initial 30 min incubation period (−5 to 30 min) highlighting the rapid early-phase membrane permeabilization kinetics following peptide addition. Vertical dashed lines indicate the time of treatment addition. Data are presented as the mean ± SD (*n* = 3).

To evaluate the effect of A34 AMP on bacterial cytoplasmic membrane integrity, membrane permeabilization kinetics were monitored over a 24 h period (Figure 7a) alongside an expanded view of the initial 30 min phase (Figure 7b) using a SYTOX Green fluorescence uptake assay across concentrations ranging from 0.25× to 4× MIC. Before AMP A34 was added (−5 to 0 min), baseline SYTOX Green fluorescence was comparable across all groups at approximately 30–38 × 10^3^ AU (Figure 7b). Upon treatment addition, fluorescence increased in both a concentration- and time-dependent manner. As shown in the expanded early-phase profile (Figure 7b), treatment with 4× MIC caused a rapid surge in fluorescence to approximately 80 × 10^3^ AU within 3 min post-addition, exceeding the initial rate of the 1% Triton X-100 positive control and remaining plateaued at 80–90 × 10^3^ AU throughout the 24 h incubation (Figure 7a). At 2× MIC, membrane permeabilization progressed more gradually during the first 30 min, reaching approximately 59 × 10^3^ AU at 30 min (Figure 7b) and 81 × 10^3^ AU at 24 h (Figure 7a). Lower concentrations (1×, 0.5×, and 0.25× MIC) exhibited slight increases during the initial 30 min window (39–49 × 10^3^ AU; Figure 7b) followed by steady, gradual rises over the 24 h period to reach approximately 77, 70, and 57 × 10^3^ AU, respectively (Figure 7a). Meanwhile, the untreated negative control remained stable at approximately 30–34 × 10^3^ AU over the entire 24 h assay (Figure 7a,b). Together, these results demonstrate a concentration- and time-dependent increase in cytoplasmic membrane permeability, with rapid early-onset membrane disruption occurring within minutes at 4× MIC.

### 3.9. Effect of Physicochemical Treatments on Antimicrobial Activity

**Figure 8:**
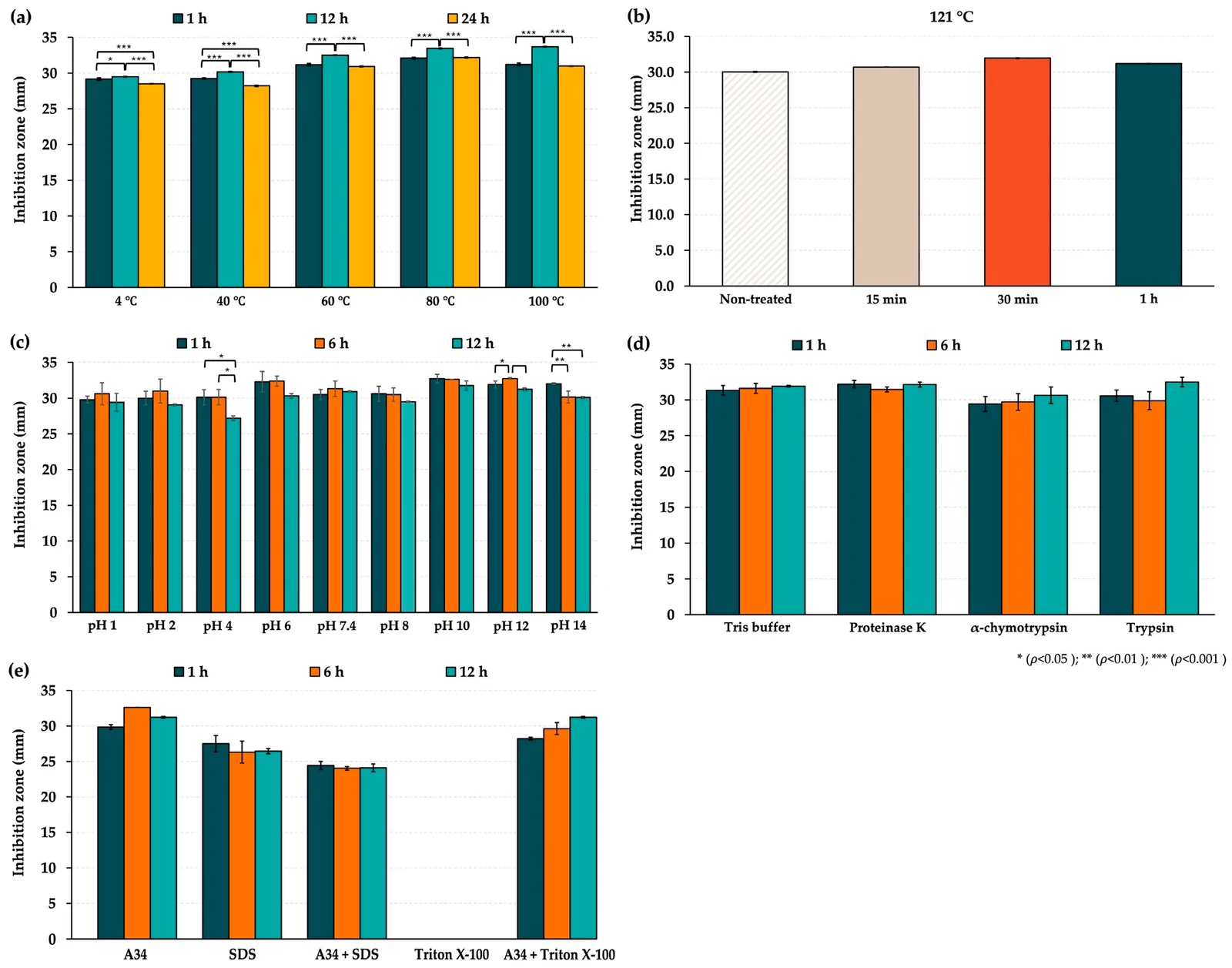
Effect of physicochemical treatments on the antimicrobial activity of A34 against MRSA strain 2468. Antimicrobial activity was evaluated by measuring the inhibition-zone diameters of A34 following exposure to (**a**) different temperatures, (**b**) autoclave, (**c**) pH conditions, (**d**) proteolytic enzymes, and (**e**) detergents.

Temperature, exposure time, and their interaction significantly affected the antimicrobial activity of A34 AMP against MRSA strain 2468. At 4–100 °C, inhibition-zone diameters changed significantly over time under most conditions. For each temperature in this range, activity at 24 h was lower than at 12 h (Figure 8). At 4 °C, the inhibition zone decreased from 29.50 ± 0.04 mm at 12 h to 28.52 ± 0.03 mm at 24 h (*p* < 0.05). At 60–100 °C, zones were larger at 12 h than at 1 or 24 h (Figure 8a). After treatment at 121 °C, inhibition zones were 30.70 ± 0.02, 31.95 ± 0.05, and 31.18 ± 0.03 mm after 15, 30, and 60 min, respectively, and differed significantly over time Rather than indicating an increase in intrinsic chemical stability or antibacterial potency, these modest increases in inhibition-zone diameter upon heating at 60–100 °C and autoclaving at 121 °C likely reflect thermally induced disaggregation of hydrophobic peptide oligomers and improved aqueous solubility, which facilitate faster lateral diffusion through the agar matrix (Figure 8b). Both pH and exposure time significantly affected activity. Activity remained similar over time under most pH conditions, but significant changes were observed at pH 4, 12, and 14. At pH 4, the inhibition zone decreased to 27.19 ± 0.31 mm after 12 h (*p* < 0.05) and was smaller than at 1 and 6 h. At 12 h, the zone at pH 4 was also smaller than those at pH 6, 7.4, 8, 10, 12, and 14. Protease treatment and exposure time had significant overall effects on antimicrobial activity, whereas the protease × time interaction was not significant. For trypsin-treated samples, the inhibition zone increased from 29.89 ± 1.25 mm at 6 h to 32.50 ± 0.68 mm at 12 h (*p* < 0.05). Because the octapeptide sequence (LLLLLLAH) lacks lysine and arginine cleavage sites, this expansion of the inhibition zone after trypsin exposure is attributable to altered peptide dispersion, disaggregation, or enzymatic digestion of trace co-eluting matrix components that enhances peptide diffusion through the agar gel, rather than an enhancement in intrinsic antimicrobial activity. Meanwhile, no significant changes in inhibition-zone diameter over time were observed following incubation with α-chymotrypsin or proteinase K (Figure 8d). Antimicrobial activity also differed among surfactant conditions at each time point. A34 AMP combined with SDS consistently produced smaller inhibition zones than A34 AMP alone, particularly at 1 and 12 h. Activity changed significantly over time in the AMP-only and AMP + Triton X-100 groups (*p* < 0.05). Triton X-100 alone did not inhibit MRSA strain 2468 (Figure 8e).

### 3.10. Biosynthetic Gene Cluster Mining and Proposed Structural Model of the Putative NRPS Assembly Line

**Figure 9:**
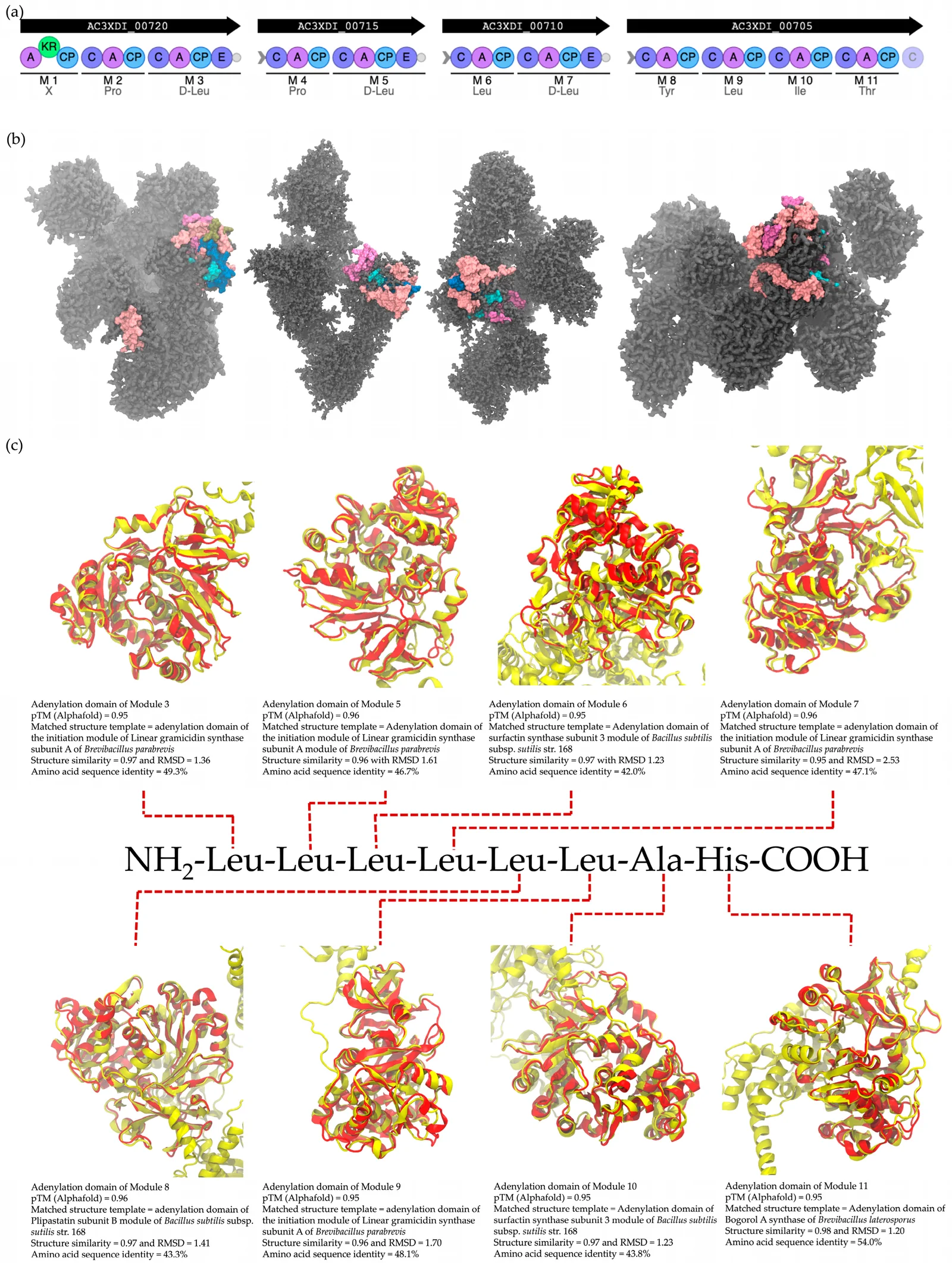
Genomic organization, three-dimensional subunit architecture, and adenylation (A) domain structural alignment of the putative 11-module nonribosomal peptide synthetase (NRPS) biosynthetic gene cluster (BGC) associated with the octapeptide LLLLLLAH in *Brevibacillus* sp. A34. (**a**) Domain organization and linear module arrangement of the 11-module NRPS cluster spanning four contiguous open reading frames (AC3XDI_00720, AC3XDI_00715, AC3XDI_00710, and AC3XDI_00705) predicted by antiSMASH. Enzymatic domains are denoted as follows: A, adenylation; C, condensation; CP, peptidyl carrier protein; E, epimerization; and KR, ketoreductase. Sequence-based monomer predictions for Modules 1 through 11 (M1–M11) are displayed beneath each module. (**b**) Three-dimensional structural models of the four multi-modular NRPS synthetase subunits encoded by AC3XDI_00720 (Modules 1–3), AC3XDI_00715 (Modules 4–5), AC3XDI_00710 (Modules 6–7), and AC3XDI_00705 (Modules 8–11), illustrating the overall protein scaffolds (grey mesh) and the spatial localization of the highlighted catalytic core regions (colored surface representations). The colors on the predicted protein structures indicate the functions of the peptide synthesis modules: pink, mauve, cyan, tan, and blue represent the adenylation domain, condensation domain, peptidyl carrier protein domain, conserved protein domain family, and epimerization domain, respectively. Gray indicates the overall protein structure. (**c**) Three-dimensional structural superposition and proposed biosynthetic mapping of the eight candidate A-domains (Modules 3 and 5–11) onto the de novo MS/MS-derived octapeptide sequence NH_2_-Leu-Leu-Leu-Leu-Leu-Leu-Ala-His-COOH. AlphaFold-predicted models (red) are superimposed onto their closest characterized NRPS structural templates (yellow), displaying predicted template modeling (pTM) scores, matched template identities, structural similarity indices, root-mean-square deviation (RMSD) values, and amino acid sequence identities.

Genome mining using antiSMASH 8.0.4 identified 23 putative biosynthetic gene cluster regions. Among these, two NRPS-associated regions showed similarity to known BGCs for laterocidine and bogorol A. The laterocidine-like region (region 1.3; 351,483–440,836 nt) showed substantial similarity in gene content and organization to the reference cluster, whereas the bogorol A-like region (region 6.2; 152,335–247,268 nt) contained several conserved biosynthetic genes. These findings indicate that strain A34 has the genomic capacity to produce nonribosomal peptide metabolites with potential antimicrobial activity. Interestingly, analysis of the NRPS-associated regions revealed a novel NRPS biosynthetic gene cluster (BGC) with no matches to previously reported NRPS architectures. Specifically, four predicted proteins of NRPS cluster contains 11 modules responsible for synthesizing an 11-amino-acid peptide, which notably includes six leucine-specific modules (Figure 9a). These genomic findings support the peptide sequence (NH_2_-Leu-Leu-Leu-Leu-Leu-Leu-Ala-His-COOH) obtained via de novo MS/MS analysis, suggesting a potential link to this predicted BGC. In addition, the Resistance Gene Identifier from the Comprehensive Antibiotic Resistance Database detected no acquired antimicrobial resistance genes in the draft genome of *Brevibacillus* sp. A34 when default curated thresholds were applied.

Further analysis of the NRPS-associated regions revealed an uncharacterized NRPS BGC with no direct matches to previously reported NRPS architectures. This cluster spans four contiguous open reading frames—AC3XDI_00720 (Modules 1–3), AC3XDI_00715 (Modules 4–5), AC3XDI_00710 (Modules 6–7), and AC3XDI_00705 (Modules 8–11)—encoding a total of 11 modules. Based on sequence-level Stachelhaus code predictions in antiSMASH, the 11 modules were initially predicted to incorporate an undetermined monomer (X, Module 1), Pro (Module 2), D-Leu (Module 3), Pro (Module 4), D-Leu (Module 5), Leu (Module 6), D-Leu (Module 7), Tyr (Module 8), Leu (Module 9), Ile (Module 10), and Thr (Module 11), alongside epimerization (E) domains in Modules 3, 5, and 7 and a ketoreductase (KR) domain in Module 1 (Figure 9a,b). To evaluate the correspondence between this 11-module BGC and the de novo MS/MS-derived octapeptide (LLLLLLAH), three-dimensional structural modeling and template alignment were performed on the individual adenylation (A) domains (Figure 9c). The structural analysis supports a biosynthetic model in which eight modules—Modules 3, 5, 6, 7, 8, 9, 10, and 11—participate in assembling the octapeptide, whereas the N-terminal modules (Modules 1 and 2) and the interspersed proline-associated Module 4 are bypassed. AlphaFold modeling of the eight candidate A-domains yielded high-confidence structures with predicted template modeling (pTM) scores ranging from 0.95 to 0.96. Structural superposition of the A-domains involved in assembling the hexaleucine core (${\mathrm{Leu}}_{1}$–${\mathrm{Leu}}_{6}$; Modules 3 and 5–9) revealed that Modules 3, 5, 7, and 9 shared the highest structural homology with the A-domain of the initiation module of linear gramicidin synthase subunit A from *Brevibacillus parabrevis*, exhibiting 46.7% to 49.3% amino acid sequence identity, structural similarity scores of 0.95 to 0.97, and root-mean-square deviation (RMSD) values of 1.36 to 2.53 Å. Furthermore, the A-domain of Module 6 matched the A-domain of surfactin synthase subunit 3 from *Bacillus subtilis* subsp. *subtilis* str. 168 (42.0% sequence identity; structural similarity = 0.97; RMSD = 1.23 Å), while the A-domain of Module 8 aligned with the A-domain of plipastatin subunit B from *B. subtilis* subsp. *subtilis* str. 168 (43.3% sequence identity; structural similarity = 0.97; RMSD = 1.41 Å). For the C-terminal region of the octapeptide, the A-domain of Module 10 (assigned to ${\mathrm{Ala}}_{7}$) aligned closely with the A-domain of surfactin synthase subunit 3 from *B. subtilis* subsp. *subtilis* str. 168 (43.8% sequence identity; structural similarity = 0.97; RMSD = 1.23 Å). Finally, the A-domain of the terminal Module 11 (assigned to ${\mathrm{His}}_{8}$) exhibited the highest sequence conservation within the cluster, aligning with the A-domain of bogorol A synthase from *Brevibacillus laterosporus* with 54.0% amino acid sequence identity, a structural similarity score of 0.98, and an RMSD of 1.20 Å. Collectively, these structural alignments support a putative biosynthetic link between this 11-module NRPS gene cluster and the isolated octapeptide LLLLLLAH.

## 4. Discussion

In this study, strain A34, isolated from mangrove sediment, showed the closest relation to *Brevibacillus laterosporus* and produced an antimicrobial fraction active against *Staphylococcus aureus*, including three MRSA strains. No activity was observed against *Escherichia coli*, *Pseudomonas aeruginosa*, or *Klebsiella pneumoniae* under the conditions tested. This selective activity may partly reflect differences in cell-envelope architecture. In Gram-negative bacteria, the lipopolysaccharide (LPS)-rich outer membrane forms an additional permeability barrier that can restrict the access of antimicrobial compounds to the cytoplasmic membrane and other cellular targets [48,49]. The physicochemical properties of the identified A34 peptide may provide an additional explanation. Although hydrophobicity can facilitate partitioning into lipid bilayers, excessive hydrophobicity may promote peptide self-association and thereby reduce the amount of freely available peptide capable of interacting with bacterial membranes [50,51]. The leucine-rich composition of A34 may further increase its tendency to undergo hydrophobic association in aqueous environments, as such interactions can drive the self-assembly of peptide-based systems [52]. Together, the Gram-negative outer-membrane barrier and the physicochemical properties of the putative peptide may restrict access of the active compound to the cytoplasmic membrane or other cellular targets. Nevertheless, this interpretation remains hypothetical and requires confirmation through direct analyses of peptide self-association, LPS binding, and outer membrane permeabilization.

Antimicrobial activity was first detected after 12 h of cultivation, remained high through 24 h, and persisted at a relatively stable level for up to 168 h. Although activity emerged during the exponential growth phase, it did not increase in parallel with cell density. This pattern suggests that production or extracellular accumulation of the active compound may be associated with a particular physiological state rather than being determined solely by biomass. A similar growth-phase-dependent production pattern has been reported for *Brevibacillus laterosporus*, in which antimicrobial activity increased during active growth and reached a plateau from the late logarithmic to early stationary phase without continuing to increase with cell density [53]. The period between 12 and 24 h may therefore represent a preliminary harvesting window for strain A34. The apparent production window of A34 was earlier than that reported or used for some other mangrove-derived bacteria. For example, *Bacillus paralicheniformis* NNS4-3 exhibited maximum anti-MRSA activity at approximately 48 h [10], whereas *Brevibacillus halotolerans* 7WMA2 was harvested after 48 h of cultivation [17]. Direct comparisons should nevertheless be made cautiously because antimicrobial production is influenced by culture-medium composition, aeration, inoculum size, nutrient availability, and other cultivation conditions. These variables should therefore be optimized specifically for strain A34.

The three-step purification procedure increased the specific activity of the A34 preparation by approximately 35-fold relative to the starting material, indicating substantial enrichment of the active component. However, the final activity yield was only 4%, suggesting considerable loss during purification. The active RPC fraction eluted at approximately 40% of the organic-solvent gradient, consistent with the retention of a compound with appreciable hydrophobic character. De novo MS/MS analysis of the RPC fraction yielded an octapeptide assignment of LLLLLLAH, with a calculated average molecular mass of approximately 905 Da for the unmodified sequence. Its high leucine content, the calculated GRAVY value was 2.675, and 87.5% of the residues were classified as hydrophobic. These properties may favor partitioning into lipid-rich regions of bacterial membranes but may also promote aggregation, poor aqueous solubility, and nonspecific interactions with mammalian cell membranes [50,54,55]. Furthermore, the α-helical conformation dynamics of the octapeptide LLLLLLAH in aqueous versus membrane environments should be carefully proposed and interpreted. Because a canonical α-helix requires 3.6 residues per turn stabilized by backbone hydrogen bonds, an eight-residue peptide can form at most two helical turns (up to four internal hydrogen bonds) and is inherently susceptible to terminal end-fraying and solvation by competing water molecules. Consequently, in bulk aqueous solution, short linear antimicrobial peptides (≤10 residues) rarely maintain a stable monomeric α-helix, instead existing as unstructured random coils or undergoing hydrophobically driven supramolecular self-assembly due to their high aliphatic content (GRAVY = 2.675) [56,57]. Moreover, deep learning predictors such as AlphaFold are trained predominantly on folded protein templates and do not explicitly account for bulk aqueous solvation, often overestimating α-helical stability in short leucine- and alanine-rich sequences [57]. Nevertheless, the short α-helical segment predicted in Figure 4b likely represents the bioactive, interface-stabilized conformation adopted when A34 AMP partitions into the hydrophobic core of the *S. aureus* cytoplasmic membrane [56,58]. Upon electrostatic and hydrophobic contact with bacterial phospholipid bilayers, short disordered peptides frequently undergo an environment-induced coil-to-helix transition—or insert as short amphipathic helical rods that self-associate within the lipid bilayer—where the low dielectric constant of the membrane interior stabilizes intramolecular backbone hydrogen bonding [56]. Additionally, as noted in our genomic analysis of the NRPS epimerization (E) domains (Modules 3, 5, and 7), if the native peptide harbors alternating D- and L-leucine residues, it may alternatively adopt a gramicidin-like β-helical or turn conformation rather than a canonical all-L α-helix [59,60]. Therefore, alongside 1D/2D NMR spectroscopy, future work will employ far-UV circular dichroism (CD) spectroscopy to experimentally characterize the secondary structure of synthetic LLLLLLAH across aqueous buffer and membrane-mimetic environments (such as 50% trifluoroethanol [TFE] and sodium dodecyl sulfate [SDS] micelles) [57].

Furthermore, a well-recognized analytical limitation of de novo peptide sequencing via conventional collision-induced dissociation (CID) or higher-energy collisional dissociation (HCD) tandem mass spectrometry (LC–MS/MS) is the inability to differentiate between the constitutional (isobaric) branched-chain isomers leucine (Leu, L) and isoleucine (Ile, I). Because both residues share an identical elemental composition (C_6_H_13_NO_2_) and monoisotopic residue mass (113.08406 Da), backbone fragmentation into *b*- and *y*-ion series alone formally defines the N-terminal hexapeptide tract as an ambiguous [L/I]_6_AH motif unless specialized high-energy side-chain fragmentation (such as *w*-ion analysis via electron-induced dissociation) or nuclear magnetic resonance (NMR) spectroscopy is performed [61,62]. In the present study, however, the assignment of this [L/I]_6_AH sequence as the hexaleucine octapeptide (LLLLLLAH) is strongly supported by two complementary lines of genomic and synthetic evidence. First, bioinformatic analysis of the candidate 11-module NRPS biosynthetic gene cluster (AC3XDI_00720–AC3XDI_00705) revealed that the Stachelhaus specificity-conferring codes of five of the six N-terminal core modules (Modules 3, 5, 6, 7, and 9) specifically predict leucine incorporation (D-Leu or L-Leu). Moreover, three-dimensional AlphaFold structural alignments confirmed that the adenylation (A) domains of these modules share high topological conservation (structural similarity = 0.95–0.97; RMSD = 1.23–2.53 Å) with experimentally characterized leucine-activating A-domains from *Brevibacillus parabrevis* linear gramicidin synthase subunit A and *Bacillus subtilis* surfactin synthase subunit 3. Second, to experimentally test this hexaleucine assignment, the pure all-leucine octapeptide was chemically synthesized via Fmoc solid-phase peptide synthesis (SPPS) and evaluated alongside the native RPC-purified fraction. The synthetic hexaleucine peptide reproduced the exact antibacterial potency and bactericidal profile of the native fraction across all tested *S. aureus* and MRSA isolates (MIC = 62 µg/mL; MBC = 62–250 µg/mL; Table 2). While definitive chemical verification of each individual side-chain spin system in the native isolate will be completed using 1D and 2D NMR spectroscopy (^1^H–^1^H COSY, TOCSY, and ^1^H–^13^C HSQC/HMBC) in our follow-up work [62,63], the convergence of A-domain substrate specificity modeling and synthetic bioactivity equivalence provides compelling support for the LLLLLLAH octapeptide assignment.

Antimicrobial susceptibility testing showed that the estimated MIC of A34 was 62 µg/mL for the A34 RPC fraction against all four *S. aureus* strains tested. The estimated MBC/MIC ratio was 1 for each of the three MRSA strains and 4 for *S. aureus* TISTR 517. These values meet the commonly applied MBC/MIC criterion of ≤4 for a bactericidal agent under the conditions tested. Although the nominal MIC of the A34 preparation was lower than that of vancomycin, this comparison should be interpreted cautiously. The concentration of A34 was calculated from the total dry weight of the RPC fraction, and neither its chemical homogeneity nor the proportion represented by the active compound has been established. The results therefore do not demonstrate that the isolated compound has greater intrinsic potency than vancomycin. Nevertheless, they indicate substantial anti-staphylococcal activity at a low nominal concentration of the RPC fraction.

The time-kill results further supported the bactericidal activity of the A34 fraction. At 2× and 4× MIC, viable counts of all four strains fell below the limit of detection within 20 h, whereas exposure to 1× MIC produced an approximately 3-log_10_ reduction in viable counts. The more rapid decline observed at the higher concentrations indicates concentration-dependent killing. The three MRSA strains appeared to be killed more rapidly than *S. aureus* TISTR 517, particularly at 1× MIC. However, because the tested strains were not isogenic and the number of isolates was small, this difference should not be attributed directly to methicillin resistance. Strain-specific differences in cell-envelope architecture, membrane lipid composition, growth rate, or the expression of stress-response systems may instead contribute to the observed variation in susceptibility [64,65,66].

The SYTOX Green and SEM findings provide complementary evidence that membrane permeabilization contributes to the antibacterial action of the A34 fraction. At 4× MIC, SYTOX Green fluorescence increased within 3 min and approached the level produced by the Triton X-100 control, indicating rapid loss of membrane exclusion of the dye. At lower concentrations, fluorescence increased more gradually, consistent with the concentration-dependent pattern observed in the time-kill assay. SEM examination further showed that treated cells developed rough and irregular surfaces, bleb-like structures, deformation, and aggregation. Comparable increases in membrane-impermeant dye uptake accompanied by morphological alterations have been reported for peptide 1018M in MRSA cells [27] and for other anti-MRSA preparations obtained from mangrove-derived microorganisms [10,12]. However, SYTOX Green uptake demonstrates increased membrane permeability but does not establish a specific pore-forming mechanism. Similarly, SEM reveals surface morphology after fixation and dehydration and cannot directly demonstrate leakage of cytoplasmic contents or exclude preparation-related artifacts. The combined findings therefore support membrane permeabilization as an important component of the antibacterial effect of A34 but do not exclude the involvement of intracellular or cell-wall-associated targets.

The physicochemical profile of A34 AMP (LLLLLLAH) necessitates a critical evaluation of its potential for off-target mammalian cytotoxicity and hemolytic activity, as well as a comparative projection of its toxicity profile based on sequence-similar antimicrobial peptides in the Antimicrobial Peptide Database (APD) [67]. Because the octapeptide comprises 75.0% leucine and 87.5% total hydrophobic residues (Leu and Ala), it exhibits a high grand average of hydropathicity (GRAVY = 2.675), an aliphatic index of 305.0, and a near-neutral net charge (+0.033) at physiological pH (7.4). In membrane-active antimicrobial peptides, excessive hydrophobicity above the optimal amphipathic window (~40–60%) frequently diminishes cell-type selectivity: whereas cationic charges drive selective electrostatic attraction toward anionic bacterial membranes rich in phosphatidylglycerol and cardiolipin, highly hydrophobic cores can partition non-specifically into zwitterionic mammalian phospholipid bilayers (enriched in phosphatidylcholine, sphingomyelin, and cholesterol) via hydrophobic interactions, leading to erythrocyte hemolysis and host-cell membrane lysis [56,59]. This biophysical trade-off is clearly illustrated by comparing LLLLLLAH against its top sequence-similar homologs identified in the APD—specifically the de novo-designed peptides GH8 (APD ID: AP05797; 55.56% sequence similarity), KL6A4 (APD ID: AP04617; 53.85% sequence similarity), and L4K2W4 (APD ID: AP05661; 50.0% sequence similarity)—as well as *Brevibacillus* nonribosomal peptides. A34 AMP octapeptides, LLLLLLAH shares the highest sequence similarity (55.56%) and C-terminal leucine–histidine (-LLXH) architecture with the de novo synthetic octapeptide GH8 (GLLWHLLH-NH_2_; 8 residues, 50% Leu, 25% His, 63% total hydrophobicity, net charge +3) and its extended 12-residue congener GH12 (GLLWHLLHHLLH-NH_2_) [68]. Circular dichroism (CD) spectroscopy demonstrated that GH8 adopts an α-helical conformation in membrane-mimetic environments (25 mM SDS micelles and 50% TFE), while replacing two hydrophobic positions with histidine residues lowers its overall hydrophobicity to 63% and prevents non-specific mammalian toxicity, as evidenced by the high viability of human gingival fibroblasts exposed to the GH peptide series [68]. The comparison between the Leu/Ala-rich peptide KL6A4 (AAKLLLKLLLKAA; 13 residues, 46% Leu, 31% Ala, 77% total hydrophobicity, net charge +4) [69] and the Leu/Lys-rich heptapeptide L4K2W4 (LLKWLLK; 7 residues, 57% Leu, 71% total hydrophobicity, net charge +3) [70] highlights a critical hydrophobicity threshold governing human red blood cell (RBC) hemolysis. In L4K2W4 (71% hydrophobicity), interrupting the two di-leucine (LL) motifs with cationic lysine residues (K) and a single interfacial tryptophan (W_4_) yields potent anti-*S. aureus* activity (MIC = 25 µg/mL) while remaining non-hemolytic against human RBCs (only 4.61% hemolysis at 200 µg/mL) [70]. Conversely, when total Leu/Ala hydrophobicity increases to 77% in KL6A4, which transitions from a random coil in aqueous PBS to an α-helix in 30 mM SDS and inhibits *S. aureus* ATCC 29213 at an MIC of 32 µM, the elevated aliphatic content induces 5% human RBC hemolysis at just 8 µM (below its anti-staphylococcal MIC) [69]. Similarly, within the genus *Brevibacillus*, linear gramicidin from *B. parabrevis*, whose initiation A-domain shares high structural homology with Modules 3, 5, 7, and 9 of the strain A34 NRPS cluster, possesses an uncharged, >85% hydrophobic core that confers strong anti-Gram-positive activity via membrane channel formation but induces marked human RBC hemolysis, restricting its clinical deployment to topical ophthalmic and dermatological formulations [59]. Based on these comparative sequence–toxicity benchmarks (L4K2W4 [71% hydrophobicity, non-hemolytic at 200 µg/mL] < KL6A4 [77% hydrophobicity, 5% hemolysis at 8 µM] < LLLLLLAH [87.5% hydrophobicity, GRAVY = 2.675]), the findings propose a differentiated toxicity profile for A34 AMP (LLLLLLAH). In its all-L synthetic configuration, because the uninterrupted hexaleucine–alanine core (LLLLLLA-) exceeds the 77% hydrophobicity threshold of KL6A4 and lacks internal cationic lysine interruptions, the peptide is predicted to exhibit moderate-to-high hemolytic potential and concentration-dependent mammalian cytotoxicity at elevated doses (with an expected hemolytic concentration possibly overlapping its anti-MRSA MIC of 62 µg/mL). Nevertheless, two intrinsic structural features of native LLLLLLAH may modulate its host-cell interactions under pathophysiological conditions. It is analogous to the histidine-rich design of GH8 (GLLWHLLH-NH_2_) [68]. However, in the absence of experimental mammalian cytotoxicity and hemolysis data. Future work should be performed to establish its selectivity index (SI = HC_50_/MIC and IC_50_/MIC) via quantitative human RBC hemolysis assays and tetrazolium-based (MTT/MTS) viability assays across mammalian cell lines (such as Vero, HEK-293 and HaCaT keratinocytes), alongside rational SAR optimization inspired by L4K2W4 and GH8 (such as targeted Leu-to-Lys or Leu-to-His substitutions to lower hydrophobicity toward ≤70%).

The A34 RPC fraction retained detectable antimicrobial activity following exposure to a broad range of physicochemical treatments. Across the temperatures tested, samples incubated for 12 h generally produced larger inhibition zones than those incubated for 1 or 24 h, although considerable activity remained after 24 h. Activity was also detected following exposure to 121 °C under the conditions tested. Similarly, the preparation retained activity after exposure to acidic and alkaline conditions, incubation with proteinase K, α-chymotrypsin, or trypsin, and treatment with SDS or Triton X-100. These findings demonstrate broad retention of functional antibacterial activity but should not be interpreted as direct evidence of chemical or structural stability. The statistically significant effects of temperature, pH, and exposure time indicate that the preparation was not entirely unaffected by these treatments. Moreover, increases or decreases in inhibition-zone diameter do not necessarily represent activation or degradation of the active compound. Heating, pH adjustment, protease treatment, and exposure to surfactants may alter the solubility, aggregation, or diffusion through agar of a hydrophobic molecule [21,71]. Similarly, the smaller inhibition zones observed in the presence of SDS may reflect interactions between the surfactant and the active material rather than direct degradation alone [69,72]. The physicochemical stability profile of A34 AMP across thermal, pH, proteolytic, and surfactant challenges must be interpreted in the context of peptide diffusion kinetics within agar [73]. While antibacterial activity was retained across wide temperature (4–121 °C) and pH (pH 2–14) ranges, heating at 60–100 °C for 12 h, autoclaving at 121 °C for 15–60 min, and incubation with trypsin for 12 h resulted in modest but statistically significant increases in inhibition-zone diameters. In agar well diffusion assays, inhibition-zone radii are governed not only by intrinsic antibacterial potency (MIC), but also by the molecular diffusion coefficient and solubility of the analyte within the aqueous agar network [73,74].

Because LLLLLLAH possesses a highly hydrophobic hexaleucine–alanine core (GRAVY = 2.675; 87.5% hydrophobic residues), it is prone to self-association into hydrophobic oligomers or supramolecular aggregates in aqueous buffer, which restricts diffusivity through crosslinked agar pores [75,76]. Exposure to high thermal energy (60–121 °C) disrupts intermolecular hydrophobic interactions, promoting thermal disaggregation into smaller, more soluble monomeric species that diffuse more rapidly through the agar matrix and thereby produce wider zones of inhibition without increasing intrinsic antimicrobial potency. Conversely, partitioning of the hydrophobic octapeptide into larger anionic SDS micelles reduces its free diffusion rate in agar, explaining the smaller inhibition zones observed in the A34 + SDS group compared with A34 alone [75,76]. Similarly, the zone expansion observed following 12 h of trypsin exposure reflects enhanced agar diffusion rather than proteolytic activation: because LLLLLLAH contains neither lysine (Lys) nor arginine (Arg) residues, it lacks canonical trypsin cleavage sites and is intrinsically resistant to tryptic degradation. Instead, a plausible mechanism is proposed wherein tryptic hydrolysis of trace co-eluting background proteins (or localized enzymatic disaggregation) liberates non-covalently bound peptide molecules, thereby increasing the fraction of freely diffusible peptide in the agar well and resulting in a larger zone of inhibition [73,77].

Whole-genome Genome BLAST Distance Phylogeny analysis placed A34 within the genus *Brevibacillus* and showed a close phylogenomic relationship with *Brevibacillus laterosporus* DSM 25, supported by a GBDP bootstrap value of 99%. This high support value indicates that the observed branching pattern was robust within the set of genomes included in the analysis. It does not, however, independently establish species-level identity. The close relationship is biologically relevant because members of the genus *Brevibacillus*, particularly *Brevibacillus laterosporus* and closely related taxa, are known to produce structurally diverse peptide-derived antimicrobial compounds [16,78].

Genome mining with antiSMASH identified 23 putative biosynthetic gene cluster regions, demonstrating the genetic potential of A34 to produce a diverse range of secondary metabolites. This number is comparable to the 23 BGCs reported in the genomes of *Brevibacillus halotolerans* 7WMA2, B-4359 and *Brevibacillus laterosporus* species. Two regions in the A34 genome also showed similarity to gene clusters associated with the biosynthesis of laterocidine and bogorol A, further supporting the established capacity of *Brevibacillus* species to produce non-ribosomal peptide antibiotics [16,79].

Laterocidine is a partially cyclic cationic lipopeptide discovered through genome mining and is active predominantly against Gram-negative bacteria [14,80]. In contrast, bogorols are cationic linear lipopeptides with activity against Gram-positive and Gram-negative bacteria and have been reported to permeabilize bacterial membranes through pore formation [79]. Interestingly, the predicted biosynthetic gene cluster contains non-ribosomal peptide synthetase (NRPS) modules that do not match previously reported architectures; specifically, previously known clusters lack modules capable of synthesizing six leucine residues within an 11-amino-acid peptide. Therefore, we propose that these unique NRPS modules are responsible for synthesizing the LLLLLLAH sequence identified via purification and de novo MS/MS analysis. Furthermore, this proposed octapeptide differs from characterized laterocidine and bogorol compounds in its length, amino acid composition, charge, molecular mass, and predicted structural organization. In addition, the A34-derived RPC fraction exhibited selective activity against *Staphylococcus* spp., whereas laterocidine is reported to act predominantly against Gram-negative bacteria. The antiSMASH results therefore indicate similarity to known biosynthetic architectures but do not demonstrate that strain A34 produced laterocidine, bogorol A, or their analogues under the culture conditions used.

While de novo MS/MS sequencing identified the active anti-staphylococcal component as the octapeptide LLLLLLAH, genome mining of *Brevibacillus* sp. A34 predicted an 11-module NRPS BGC spanning open reading frames AC3XDI_00720 to AC3XDI_00705. Although canonical NRPS megasynthases follow a strict colinearity rule, where each module incorporates a single building block via a thiotemplate mechanism, comparative biosynthetic and probabilistic genome-mining analyses confirm that NRPS assembly lines frequently deviate from linear gene order through module skipping, alternative initiation, and post-translational proteolytic processing [81]. To reconcile the 11-module architecture with the isolated 8-residue peptide, three-dimensional modeling of the four NRPS subunits and template alignment of the individual A-domains established a biosynthetic model in which eight functional modules (Modules 3 and 5–11) synthesize the octapeptide (LLLLAH), while the initial two modules (Modules 1 and 2) and the interspersed proline-associated Module 4 are omitted. First, the omission of the proline-predicted Module 4 (AC3XDI_00715) between the leucine-activating Module 3 (AC3XDI_00720) and Module 5 (AC3XDI_00715) is supported by documented module-skipping and non-colinear assembly phenomena in microbial NRPS and hybrid polyketide synthase (PKS)–NRPS systems [81,82,83]. In bacterial systems, structural analyses of Bacillus lipopeptide assembly lines indicate that variations in inter-subunit interfaces—such as altered or absent communication-mediating (COM) domains in the bacillomycin D synthetase subunit BamB—can trigger direct module skipping during peptide elongation [84]. Similarly, metabolic profiling of deep-sea Bacillus isolates producing the nobilamide family of NRP-derived peptides demonstrated that single NRPS loci routinely yield truncated peptide congeners through non-canonical skipping pathways [82] while genome-guided characterization of hybrid PKS–NRPS assembly lines has confirmed that 14-module synthases naturally synthesize shortened 11-residue peptides via internal module skipping [83]. Mechanistically, this process parallels the native biosynthesis of the myxobacterial lipopeptide myxochromide S, in which an inactive Module 4 is completely skipped during peptide core assembly [85], as well as domain-deletion studies on the plipastatin NRPS in *Bacillus subtilis* [86]. In the plipastatin system, functional perturbation of an internal A-domain induced conformational flexibility within the multi-enzyme complex; rather than halting elongation, the upstream peptidyl intermediate bypassed adjacent modules, including a proline-activating module, via direct carrier-protein-to-carrier-protein transfer, allowing downstream domains to resume elongation and release shortened octapeptide and hexapeptide derivatives [86]. Furthermore, comparative analyses of 2051 bacterial NRPS A-domains across 67 species have shown that sequence divergence and domain recombination within proline- and aliphatic-activating modules frequently alter canonical substrate loading and inter-domain coordination [87]. In *Brevibacillus* sp. A34, Module 4 resides directly at the N-terminal inter-subunit junction of AC3XDI_00715 immediately following the E-domain of Module 3 (AC3XDI_00720). Spatial constraints at this subunit interface, or relaxed substrate gating of the adjacent condensation (C) and peptidyl carrier protein (CP) domains, may facilitate the direct transfer of the leucine residue intermediate from the CP domain of Module 3 to Module 5, bypassing Module 4 entirely [84,88]. Second, the absence of residues corresponding to Modules 1 and 2 in the mature octapeptide may be explained by either post-translational proteolytic truncation or direct biosynthetic initiation at Module 3. Module 1 harbors an N-terminal ketoreductase (KR) domain and an unassigned monomer specificity (X); in bacterial NRPS pathways, upstream modules frequently assemble an N-terminal leader sequence or pro-peptide cap to prevent intracellular self-toxicity, which is subsequently cleaved by cellular peptidases upon secretion to release the mature, active peptide. Alternatively, as observed in truncated assembly lines of engineered plipastatin synthetases [86], impaired catalytic communication between Modules 2 and 3 could uncouple upstream synthesis, allowing Module 3, whose A-domain shares high structural homology with the initiation module of linear gramicidin synthase subunit A in *Brevibacillus parabrevis* (structural similarity = 0.97; RMSD = 1.36 Å), to function independently as the primary initiation module. Third, three-dimensional structural modeling reconciles the sequence-level Stachelhaus prediction discrepancies observed in Modules 8, 10, and 11. While sequence-only algorithms initially suggested Tyr (Module 8), Ile (Module 10), and Thr (Module 11), AlphaFold structural alignments (pTM = 0.95–0.96) revealed high topological conservation with characterized A-domains from *Bacillus subtilis* plipastatin subunit B (Module 8; RMSD = 1.41 Å), *Bacillus subtilis* surfactin synthase subunit 3 (Module 10; RMSD = 1.23 Å), and *Brevibacillus laterosporus* bogorol A synthase (Module 11; RMSD = 1.20 Å). As demonstrated in Bacillus lipopeptide synthetases, A-domains and their adjacent C-domains frequently exhibit relaxed substrate specificity, enabling the accommodation and transfer of alternative aliphatic or polar residues [86,87,88]. Fourth, the mechanism of linear peptide chain release from the megasynthase complex must be considered, as the terminal Module 11 (AC3XDI_00705) lacks a canonical *cis*-acting Type I thioesterase (TE) or terminal reductase (TD) subdomain and instead terminates with an extra-modular C-terminal condensation (C) domain (Figure 9a). Although integrated C-terminal TE domains represent the classical termination route in bacterial NRPSs, TE-independent chain liberation is well established across non-canonical microbial NRPS pathways [84,89]. Specifically, the standalone C-terminal C-domain immediately downstream of the Module 11 CP domain may function analogously to a chain-terminating condensation ($C_{T}$) domain, utilizing its conserved active-site histidine motif to catalyze water-mediated nucleophilic hydrolysis of the octapeptidyl-S-CP thioester intermediate [84,89]. Alternatively, chain release may be catalyzed in trans by a discrete Type II thioesterase (TEII) or standalone α/β hydrolase encoded within the genome, which recognizes the stalled or completed octapeptidyl-S-CP intermediate on Module 11 and hydrolyzes the thioester linkage to release the free carboxylic acid product [89,90]. Furthermore, the incorporation of a C-terminal histidine residue at Module 11 may facilitate substrate-assisted thioester cleavage, wherein the nucleophilic imidazole side chain promotes intramolecular or water-assisted hydrolysis of the labile 4′-phosphopantetheine thioester bond. Finally, although sample degradation during multi-step chromatographic purification or MS/MS ionization could theoretically generate a truncated peptide fragment, the robust stability of the bioactive fraction across thermal, pH, protease, and surfactant treatments argues against random chemical hydrolysis, supporting a programmed biosynthetic skipping and maturation mechanism.

Nevertheless, several limitations of the present study must be acknowledged and addressed in future investigations. First, the proposed biosynthetic link between the 11-module NRPS cluster (AC3XDI_00720–AC3XDI_00705) and the octapeptide LLLLLLAH is currently based on in silico genome mining and three-dimensional structural homology modeling, where the moderate amino acid sequence identity (42.0–54.0%) across individual A-domains and the absence of a canonical *cis*-acting Type I thioesterase (TE) domain preclude definitive functional assignment without experimental validation. Second, because de novo MS/MS sequencing cannot resolve α-carbon chirality or distinguish isobaric leucine and isoleucine side chains, the exact stereochemical configuration of the native octapeptide—specifically the putative D-Leu_1_, D-Leu_2_, and D-Leu_4_ assignments predicted from the epimerization domains of Modules 3, 5, and 7, remains to be chemically confirmed [61,91]. Third, because bioactivity assays were conducted using chromatographically purified native fractions, trace co-eluting metabolites could potentially influence the observed antimicrobial potency, necessitating chemical synthesis of the pure peptide to validate its intrinsic MIC against *S. aureus* [63]. Consequently, our future work will follow a two-tiered biochemical and synthetic roadmap. To verify the biosynthetic pathway and module-skipping mechanism, we will perform: (i) transcriptional profiling (RT-qPCR and RNA-seq) to confirm co-transcription of the four NRPS open reading frames under antimicrobial-producing conditions; (ii) targeted gene disruption and in-frame domain deletions (via CRISPR-Cas9 or homologous recombination) of Module 3, the bypassed Module 4, the terminal Module 11, and the C-terminal condensation domain; (iii) in vitro ATP–PPi exchange assays on heterologously expressed A-domains (Modules 8, 10, and 11); and (iv) stable-isotope-labeled precursor feeding coupled with LC–MS/MS. Concurrently, to resolve the stereochemical configuration and validate the intrinsic antibacterial activity, we will subject the native peptide to one- and two-dimensional nuclear magnetic resonance (1D ^1^H and ^13^C NMR, along with 2D ^1^H–^1^H COSY, TOCSY, NOESY/ROESY, ^1^H–^13^C HSQC, and HMBC) spectroscopic elucidation and *J*-based configuration analysis to distinguish isobaric leucine/isoleucine spin systems and establish the exact planar connectivity and relative L/D-stereochemical configuration of each residue [61,62], followed by Fmoc solid-phase peptide synthesis (SPPS) of both the all-L octapeptide and the predicted D-Leu1,2,4 diastereomer to benchmark their intrinsic MICs, proteolytic stabilities, membrane-disruptive mechanisms, and hemolytic profiles against *S. aureus* and MRSA isolates [58,59,63].

Several limitations of the present study should be addressed in future investigations. Although the active fraction was enriched through multi-step purification and its primary sequence bioactivity was verified via chemical synthesis, the exact stereochemical configuration (D/L-chirality) of the native peptide remains tentative, and targeted genetic or biochemical validation is still required to definitively link the active compound to its putative nonribosomal peptide biosynthetic gene cluster. Furthermore, antibacterial susceptibility was evaluated against a relatively limited panel of staphylococcal strains, and the membrane permeabilization assay was performed using only a single MRSA isolate. Experimental assessments of mammalian cytotoxicity, human erythrocyte hemolysis, serum stability, antibiofilm activity, and in vivo efficacy were also beyond the scope of the current work. Provided that A34 AMP demonstrates a favorable selectivity index and retains its antibacterial potency and stability across these preclinical benchmarks, it would warrant further optimization and development as a bioactive antimicrobial scaffold.

## 5. Conclusions

This study identified the mangrove-derived isolate A34 as *Brevibacillus* sp. A34 and demonstrated that it produces an antimicrobial fraction with selective activity against *Staphylococcus aureus*, including three MRSA strains. Following a three-step purification process, the active reversed-phase chromatography (RPC) fraction exhibited an MIC of 62 µg/mL against all four tested *S. aureus* strains. The MBC/MIC ratios and time-kill kinetics supported a concentration-dependent bactericidal mode of action. Rapid SYTOX Green uptake and the morphological alterations observed by scanning electron microscopy (SEM) further indicated that membrane permeabilization contributes to the antibacterial activity of the A34 fraction. Moreover, the retention of antimicrobial activity across diverse physicochemical treatments, including thermal, pH, proteolytic, and surfactant challenges, demonstrates functional robustness under the tested conditions. De novo LC–MS/MS sequencing yielded an octapeptide assignment of LLLLLLAH, whereas genome mining identified 23 putative biosynthetic gene clusters, including regions resembling the laterocidine and bogorol A clusters, as well as a candidate 11-module NRPS cluster. However, these predicted biosynthetic gene clusters require further experimental investigation to confirm their direct role in synthesizing the active metabolite. Future studies should therefore focus on resolving the stereochemical configuration of the native peptide and functionally validating the proposed NRPS biosynthetic pathway. Comprehensive evaluations of human erythrocyte hemolytic activity, mammalian-cell cytotoxicity, serum stability, and in vivo efficacy will also be required. Collectively, these findings indicate that *Brevibacillus* sp. A34 is a promising source of anti-staphylococcal metabolites and provide a foundation for further chemical, genomic, and biological characterization of its bioactive octapeptide scaffold.

## Figures and Tables

**Table 1:** Purification balance sheet of antimicrobial compound produced by strain A34.

Purification Step	Volume (mL)	Total Dried Weight (mg)	Activity (AU/mL)	Total Activity (AU)	Specific Activity (AU/mg)	Purification Factor	Yield (%)
Cell free supernatant	987.00	9458.75	640.00	631,680.00	66.78	1.00	98.70
50% ammonium sulfate precipitation	198.50	1451.70	2560.00	508,160.00	350.05	5.24	79.40
CIEX active fraction	74.30	252.37	2560.00	190,208.00	753.68	11.29	29.72
RPC-purified A34 fraction	20.00	11.00	1280.00	25,600.00	2327.27	34.85	4.00

**Table 2:** Minimum inhibitory concentration (MIC) and minimum bactericidal concentration (MBC) values of the RPC-purified A34 fraction, chemically synthesized A34 peptide, and vancomycin against *S. aureus* TISTR 517 and three MRSA strains.

	Parameter (µg/mL)	*S. aureus* TISTR 517	MRSA Strain 142	MRSA Strain 1096	MRSA Strain 2468
RPC purified fraction of A34 peptide	Apparent MIC	62	62	62	62
	Apparent MBC	250	62	62	62
Synthetic A34 peptide	MIC	62	62	62	62
	MBC	250	62	62	62
Vancomycin	MIC	2	2	2	2
	MBC	2	2	2	2

## Data Availability

Data are available within the article. Reasonable inquiries for additional information can be directed to the corresponding author.
